# Cooperation between SMYD3 and PC4 drives a distinct transcriptional program in cancer cells

**DOI:** 10.1093/nar/gkv874

**Published:** 2015-10-10

**Authors:** Jin-Man Kim, Kyunghwan Kim, Thomas Schmidt, Vasu Punj, Haley Tucker, Judd C. Rice, Tobias S. Ulmer, Woojin An

**Affiliations:** 1 Department of Biochemistry and Molecular Biology, University of Southern California, Norris Comprehensive Cancer Center, Los Angeles, CA 90033, USA; 2 Department of Biology, College of Natural Sciences, Chungbuk National University, Cheongju, Chungbuk 361-763, Republic of Korea; 3 Department of Biochemistry and Molecular Biology, Zilkha Neurogenetic Institute, Keck School of Medicine, University of Southern California, 1501 San Pablo Street, Los Angeles, CA 90033, USA; 4 Department of Medicine, Norris Comprehensive Cancer Center, 1450 Biggy Street, Los Angeles, CA 90033, USA; 5 University of Texas at Austin, Institute for Cellular and Molecular Biology, Austin, TX 78712, USA

## Abstract

SET and MYND domain containing protein 3 (SMYD3) is a histone methyltransferase, which has been implicated in cell growth and cancer pathogenesis. Increasing evidence suggests that SMYD3 can influence distinct oncogenic processes by acting as a gene-specific transcriptional regulator. However, the mechanistic aspects of SMYD3 transactivation and whether SMYD3 acts in concert with other transcription modulators remain unclear. Here, we show that SMYD3 interacts with the human positive coactivator 4 (PC4) and that such interaction potentiates a group of genes whose expression is linked to cell proliferation and invasion. SMYD3 cooperates functionally with PC4, because PC4 depletion results in the loss of SMYD3-mediated H3K4me3 and target gene expression. Individual depletion of SMYD3 and PC4 diminishes the recruitment of both SMYD3 and PC4, indicating that SMYD3 and PC4 localize at target genes in a mutually dependent manner. Artificial tethering of a SMYD3 mutant incapable of binding to its cognate elements and interacting with PC4 to target genes is sufficient for achieving an active transcriptional state in SMYD3-deficient cells. These observations suggest that PC4 contributes to SMYD3-mediated transactivation primarily by stabilizing SMYD3 occupancy at target genes. Together, these studies define expanded roles for SMYD3 and PC4 in gene regulation and provide an unprecedented documentation of their cooperative functions in stimulating oncogenic transcription.

## INTRODUCTION

SET and MYND domain-containing proteins (SMYD) are a special class of protein lysine methyltransferases involved in methylation of histones and non-histone proteins (1–3). In human cells there are five members of the SMYD protein family, SMYD1–5, which share a distinctive architecture of their SET domain, split into two parts by a Myeloid-Nervy-DEAF1 (MYND) domain (4–6). SMYD3, which is the major focus of the current study, mediates tri-methylation of histone H3 at lysine 4 (H3K4me3) and activates transcription of a set of downstream genes containing a specific DNA motif, 5′ - CCCTCC - 3′ (2). The MYND domain of SMYD3 is highly positively charged and likely contributes to the binding of SMYD3 to target sites (5,7). SMYD3 may regulate chromatin remodeling and gene transcription by interacting with other transcription factors. In support of this idea, it has been shown that SMYD3 interacts with RNA polymerase II and RNA helicase HELZ and promotes gene expression by facilitating transcriptional elongation (2,8). Our previous work also showed that SMYD3 directly interacts with estrogen receptor (ER) and up-regulates ER target genes via H3K4me3 (9). Besides its H3K4me3-dependent function, SMYD3 also modifies non-histone proteins to regulate specific cellular reactions as exemplified by MAP3K2 methylation (1). SMYD3 has been regarded as an important factor in cancer, based on the fact that high level expression of SMYD3 has cell proliferative effects and up-regulates a number of genes involved in cell growth and proliferation (1,2,10–12). Further support for such an oncogenic function of SMYD3 is provided by studies demonstrating that suppression of SMYD3 expression by RNAi or other inhibitory reagents induces apoptosis and inhibits cell proliferation (2,13,14).

The human positive coactivator 4 (PC4) is a multifunctional protein which plays a regulatory role in diverse cellular processes, including RNA polymerase II transcription, replication, heterochromatinization and DNA repair (15–18). In transcription reactions, PC4 interacts with unwinding DNA, the basal transcription machinery and gene-specific transcription factors (15,19,20). PC4 positively regulates transcription by enhancing the effects of gene-specific activators at the initiation levels and stimulating subsequent promoter escape (21,22). PC4 does not have any chromatin remodeling and histone modification activities, but is involved in the regulation of chromatin dynamics (16–18,23). Besides serving as a transcription regulator, PC4 has been reported to have additional functions in regulating DNA end joining and repair through its single stranded DNA binding activity (16,18). By interacting with DNA repair protein XPG, PC4 could also prevent spontaneous and induced oxidative mutagenesis (16). However, in contrast to the proposed tumor suppressive function, PC4 has recently been linked to cancer, as its aberrant expression has been reported in several human cancers and cancer cell lines (24,25). A role for PC4 in cancer development is further supported by the demonstration that PC4 expression in normal dermal fibroblasts drives the tumorigenic transformation of the cells (25). However, the molecular mechanisms underlying these phenomena are poorly understood.

In this study, we employed a combination of gene expression profiling, transcription assays, ChIP-qPCR and CRISPR/dCas9 system to investigate a possible functional interaction between SMYD3 and PC4. Our data show that SMYD3 colocalizes with PC4 at genes regulating cell proliferation and invasion and establishes transcriptional competence in bladder and colon cancer cells. SMYD3 and PC4 execute transcriptional programs in a cooperative manner, as shRNA-mediated knockdown of either SMYD3 or PC4 inhibits target gene transcription and cell proliferation/invasion. Furthermore, CRISPR/dCas9-based tethering of SMYD3 mutant deficient in PC4 interaction and DNA binding to target genes is sufficient to mediate transcriptional activation, strongly suggesting that the functional contribution of PC4 to SMYD3 transactivation is mainly through the enhancement of SMYD3 occupancy at target genes.

## MATERIALS AND METHODS

### Cell lines, constructs and antibodies

HT29, T24 and RT4 cells were maintained in McCoy's 5A medium containing 10% fetal bovine serum (FBS). HCT116, CaCO2, J82, MCF7, MDA-MB-231, DU145 and UROtsa cells were maintained in Dulbecco's modified Eagle's medium (DMEM) supplemented with 10% FBS (HCT116, CaCO2, J82, MCF7, MDA-MB-231 and DU145 cells) or 5% FBS (UROtsa cells). LNCaP and MLC cells were maintained in RPMI culture media and T medium with 10% FBS, respectively. MCF-10–2A cells were grown in a 1:1 mixture of DMEM and Ham's F12 supplemented with 20 ng/ml epidermal growth factor, 100 ng/ml cholera toxin, 0.01 mg/ml insulin, 500 ng/ml hydrocortisone and 5% horse serum. CCD-18Co cells were cultured in Eagle's Minimum Essential Medium with 10% FBS. For mammalian expression of SMYD3 and PC4, their cDNAs were amplified by PCR and ligated into the correct reading frames of lentiviral expression vector pLenti-Hygro (Addgene) containing FLAG coding sequences. To generate mutant SMYD3/PC4 expression vectors, SMYD3 and PC4 cDNAs were mutated by the QuikChange® II site-directed mutagenesis kit (Agilent Technologies) before the construction. For bacterial expression of wild-type and mutant versions of SMYD3 and PC4, the corresponding cDNAs were amplified by PCR and inserted into pGEX-4T1 and pET15b vectors. For mammalian expression of dCas9-FLAG/dCas9-FLAG-SMYD3 wild-type and mutant, the nuclease-deficient dCas9 (D10A and H840A) cDNA (NotI-dCas9-EcoRI) was PCR-amplified, and ligated into pIRES vector digested by NotI and EcoRI. Wild-type or mutant SMYD3 coding sequences were excised from a vector and cloned into the EcoRI-BamH1-digested pIRES-dCas9 vector. For sgRNA expression vectors, oligonucleotides listed in Supplementary Table S2 were annealed and ligated into BsmBI-digested pMLM3636 vector (Addgene). Targeting specificity and the extent of potential off-target activity of dCas9-FLAG-SMYD3 were analyzed by using TagScan (http://www.isrec.isb-sib.ch/tagger), which is a web portal to provide tools for finding short exact matches in large sequence databases (26). Further details of plasmid constructions are available upon request. Antibodies used in this study are as follows: SMYD3, HSP90A, H3K4me1, H2B and H3 antibodies from Abcam, H3K4me2 antibody from Millipore, Actin and FLAG antibodies from Sigma, SPT16 and Nucleolin antibodies from Santa Cruze, PARP1 and H3K4me3 antibodies from Active Motif and PC4 antibody from Bethyl Laboratories.

### RNA interference

For the depletion of SMYD3 and PC4, DNA oligonucleotides encoding shRNAs specific for SMYD3 3′ UTR region (5′-GCTGTGTGAACCTCTCTTATT-3′) and PC4 mRNA coding region (5′-GAACAGATTTCTGACATTGAT-3′) were annealed and ligated into the lentiviral expression vector pLKO.1 (Addgene). Lentivirus particles were generated in 293T cells by co-transfecting plasmids encoding VSV-G, NL-BH and the shRNAs. HCT116, CaCO2, J82 and T24 cells were infected by these viruses, and were selected with 2 μg/ml puromycin (Sigma) for two weeks.

### Gene expression microarray, qRT-PCR and ChIP-qPCR

Total RNA was isolated from two biological replicates of mock- or SMYD3-depleted HCT116 cells using the TRIzol reagent according to the manufacturer's instructions (Invitrogen). Gene expression microarray experiments were conducted using a whole-genome expression array (Human HT-12 v4 Expression BeadChip, Illumina). Differential gene expression was detected by using the ArrayPipe software (www.pathogenomics.ca/arraypipe). Genes showing detection *P* < 0.05 with fold change >2 were functionally analyzed in the context of gene ontology by using Ingenuity pathway software (IPA; www.ingenuity.com). For qRT-PCR, total RNA was isolated as for microarray and subjected to reverse transcription using the iScript cDNA Synthesis Kit (Bio-Rad) and the PerfeCta SYBR Green FastMix (Quanta biosciences) as recently described (27). Gene set enrichment analysis (GSEA) was performed using GSEA (http://www.broad.mit.edu/gsea; Broad Institute, Cambridge, MA) as detailed previously (28). The primers used for qRT-PCR are listed in Supplementary Table S3. ChIP assays with HCT116 cells were performed using the ChIP assay kit (Millipore) as recently described (29). Antibodies specific to SMYD3, PC4, H3K4me1, H3K4me2, H3K4me3 and H3 were used for immunoprecipitation. Immunoprecipitated DNA was analyzed with the primers that amplify the different regions of the *FNBP1, MFGE8, PDLIM7, WNT3A, SMUG1* and *KRT81* loci. The primers used for qPCR are summarized in Supplementary Table S4.

### Cell proliferation and invasion assays

HCT116 cells were incubated with 0.5 mg/ml MTT (2-[4,5-dimethylthiazol-2-yl]-2,5-di-phenyltetrazolium bromide) (Sigma) for 2 h at 37°C. The MTT formazan was dissolved in 200 μl of DMSO. The absorbance of the solution was quantified at 570 nm against 650 nm by a microplate reader (Bio-Rad). For cell invasion assays, cells were harvested and suspended in culture medium containing 5% FBS and then seeded to the upper chamber coated with Matrigel (BD Biosciences). Cells were allowed to invade toward 10% FBS in the lower chamber for 48 h. The invaded cells on the underside of the transwell filters were fixed with 10% formaldehyde for 15 min and stained with 1% crystal violet for 1 h. Cells were photographed and counted.

### Purification and identification of SMYD3-interacting partners

HCT116 cells continuously expressing FLAG-HA-SMYD3 were generated by stable transfection of pIRES-FLAG-HA-SMYD3. Nuclear extracts were prepared as described (30), and ectopic SMYD3 and its interacting proteins were purified by sequential immunoprecipitation using anti-FLAG M2 and anti-HA antibodies (Sigma) in the precipitation buffer (20 mM Tris-HCl, pH 7.3, 300 mM KCl, 0.2 mM EDTA, 20% glycerol and 0.1% Nonidet P-40). The purified proteins were resolved by 4–20% gradient SDS-PAGE, and stained with Coomassie blue. Major protein bands were excised and analyzed by liquid chromatography-tandem mass spectrometry (LC-MS/MS).

### Histone methyltransferase assays

293 cells were transfected with dCas9-FLAG plasmid or plasmids expressing dCas9-FLAG-SMYD3 wild-type or mutant for 48 h, and the proteins were purified with anti-FLAG antibody. Recombinant histone octamers (1 μg) were incubated with immunoprecipitated dCas9-FLAG or dCas9-FLAG-SMYD3 for 1 h at 30°C in HMT reaction buffer (100 mM HEPES, pH 7.8, 300 mM KCl, 2.5 mM EDTA, 25 mM dithiothreitol and 50 mM sodium butyrate) in the presence of 50 μM S-adenosyl-methionine. Histone proteins were resolved by 15% SDS-PAGE, and analyzed by immunoblotting with anti-H3K4me3 antibody.

### Protein-protein interactions

For *in vitro* interaction studies with SMYD3, His-tagged SMYD3 wild-type and mutants were synthesized by using TNT-Quick-coupled transcription/translation system (Promega), and incubated overnight with GST-PC4 immobilized on glutathione-Sepharose beads (Amersham Biosciences) at 4°C in binding buffer (20 mM Tris-HCl, pH 7.3, 0.2 M KCl, 0.2 mM EDTA, 20% glycerol and 0.01% Nonidet P-40). After washing the beads three times with the binding buffer, the beads were subjected to SDS-PAGE and immunoblotting with anti-His antibody. For binding assays with PC4, GST-PC4 wild-type and mutants were prepared from *E. coli* and incubated with His-tagged SMYD3. For co-immunoprecipitation assays, FLAG-tagged SMYD3 proteins were expressed in HCT116 cells, and whole cell lysates were prepared from the cells with lysis buffer (50 mM Tris-HCl, pH 8.0, 150 mM NaCl, 1 mM EDTA, 0.5% sodium deoxycholate, 0.1% SDS and 1% Nonidet P-40). The cell lysates were mixed with anti-FLAG antibody-conjugated Sepharose beads (Sigma) and incubated overnight with gentile rotation at 4°C. After removing the supernatant, the sample pellets were analyzed by immunoblot analysis using anti-PARP1, anti-SPT16, anti-Nucleolin, anti-H2B and anti-PC4 antibodies. To co-immunoprecipitate endogenous SMYD3 and PC4, HCT116 cell lysates were incubated with anti-SMYD3 antibody or normal rabbit IgG for overnight. After centrifugation, immunocomplexes in the supernatants were precipitated with Protein A/G-Sepharose (Santa Cruz Biotechnology) and separated on SDS-PAGE. Immunoblot analyses were performed with anti-PC4 and anti-SMYD3 antibodies.

### DNA binding assays

For in vitro binding assays, streptavidin-coated 96well plates were incubated with biotin-conjugated oligonucleotides containing three copies of SMYD3 binding sites for 2 h at room temperature. The sequences of oligonucleotides were: 5′- CGTATTCCCTCCATACTCGCGTATTCCCTCCATACTCGCGTATTCCCTCCATACTCG -3′-biotin (sense) and 5′- CGAGTATGGAGGGAATACGCGAGTATGGAGGGAATACGCGAGTATGGAGGGAATACG -3′-biotin (antisense). The plates were washed three times with wash buffer (20 mM Tris, pH 7.2, 150 mM NaCl, 0.1% BSA and 0.05% Tween-20), and incubated with SMYD3 wild-type or mutant in binding buffer (20 mM Tris, pH 7.2, 150 mM NaCl, 0.4 mM EDTA, 0.5 mM DTT, 12% glycerol and 1 μg/μl poly dI/dC) for 1 h at room temperature with gentle rotation, added PC4 wild-type or mutant, and then further incubated for 1 h at room temperature. After washing with wash buffer, the plates were incubated with FLAG/His antibodies for 1 h and then horseradish peroxidase-conjugated secondary antibody for 0.5 h. TMB substrate (Pierce) was used for color development at 450 nm, and absorbance was read by a Plate Chameleon V plate reader.

## RESULTS

### SMYD3 activates genes governing cell proliferation and invasion in cancer cells

As SMYD3 is often overexpressed in cancer cells, we first examined SMYD3 levels in bladder, breast, colon and prostate cell lines by immunoblotting. Three bladder (J82, T24 and RT4) and three colon (HCT116, CaCO2 and HT29) cancer cell lines expressed significantly higher levels of SMYD3 compared to their normal counterparts (UROtsa and CCD-18Co) (Supplementary Figure S1A). In contrast, higher expression of SMYD3 was not evident in breast (MCF7 and MDA-MB-231) and prostate (LNCaP and DU145) cancer cell lines compared to normal cell lines (MCF-10–2A and MLC). The observed overexpression of SMYD3 in bladder and colon cancer cell lines encourages the possibility that SMYD3 may facilitate key tumorigenic processes such as cell proliferation and invasion. To test this possibility, we depleted SMYD3 in two bladder (J82 and T24) and two colon (HCT116 and CaCO2) cancer cells expressing high levels of SMYD3. In this study, it was important that SMYD3 is depleted for prolonged periods, as this allows the study of progressive alterations of cell proliferation and invasion under identical conditions. This was achieved by using a lentiviral shRNA infection system. Immunoblot analysis confirmed that stable transfection of SMYD3 shRNA efficiently silenced the expression of SMYD3 in the cancer cells (Supplementary Figure S1B). MTT assays over a period of 4 days reproducibly showed that the bladder and colon cancer cells proliferate much more slowly following the depletion of endogenous SMYD3 (Supplementary Figure S1C). SMYD3 knockdown also led to a significant decrease in cell invasion compared with mock-depleted control cells (Supplementary Figure S1D). These observations are consistent with the hypothesis that SMYD3 is one of the key players stimulating proliferation and invasiveness of bladder and colon cancer cells.

**Figure 1.**
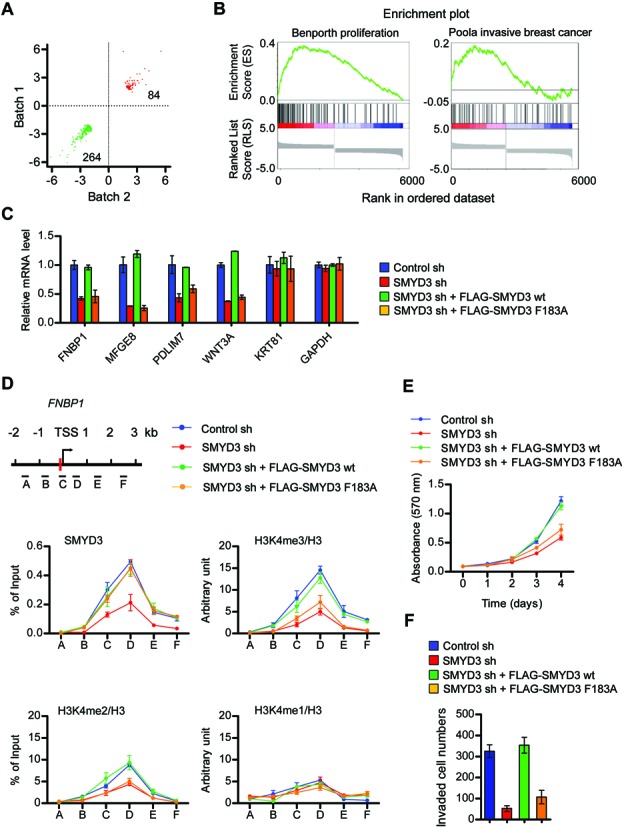
SMYD3 knockdown suppresses cell proliferation/invasion-related genes. (**A**) Control or SMYD3-depleted HCT116 cells were subjected to microarray analysis. Dots in the two-dimensional scatter-plot represent expression values for the genes with a folder change >2 in either of two independent analyses (Batch #1 and Batch #2). These analyses identified 264 genes that were down-regulated (indicated by green dots) and 84 genes that were up-regulated (indicated by red dots) in the SMYD3-depleted cells, compared to the control cells. (**B**) Summary of GSEA of microarray data. The *Y*-axis plots the enrichment score and *X*-axis is the rank of genes differentially expressed in SMYD3-depleted cells. Bar codes below enrichment plots show the rank position of individual genes differentially expressed in SMYD3-depleted cells. (**C**) SMYD3 wt and SMYD3 F183A were expressed in SMYD3-depleted HCT116 cells, and relative mRNA levels of the four down-regulated (*FNBP1, MFGE8, PDLIM7* and *WNT3A*) and two unaffected (*KRT81* and *GAPDH*) genes in SMYD3-depleted HCT116 cells were quantified by qRT-PCR. (**D**) ChIP-qPCR assays were performed in control or SMYD3-depleted HCT116 cells complemented with SMYD3 wt or SMYD3 F183A by using SMYD3, H3K4me1, H3K4me2, H3K4me3 and H3 antibodies. Precipitation efficiencies relative to non-enriched input samples were determined for the six locations across the *FNBP1* locus by qPCR with primers depicted on the left and listed in Supplementary Table S4. Percent input is determined as the amount of immunoprecipitated DNA relative to input DNA. The enrichment of H3K4me1/me2/me3 was calculated as the ratio of anti-H3K4me1/me2/me3 ChIP to anti-H3 which is an indication of local nucleosome density. Error bars represent the SD obtained from three independent experiments. The vertical red bar represents the putative SMYD3 binding site. (**E**) SMYD3-depleted HCT116 cells were infected with SMYD3 wt or SMYD3 F183A as in Supplementary Figure S3A, and changes in cell proliferation rates were measured by the MTT colorimetric assay every day for 4 days post-infection. (**F**) SMYD3-depleted HCT116 cells were complemented with SMYD3 wt or SMYD3 F183A, and cell invasion activity was determined by the Matrigel invasion assay.

To assess the functional contributions made by SMYD3 in the above results, we performed comprehensive microarray analysis using total RNA isolated from mock- or SMYD3-depleted HCT116 colon cancer cells. With a fold-change cutoff of 2.0 and *P*-value cutoff of 0.005, the gene expression profiling identified 264 genes whose expression was decreased in response to SMYD3 knockdown (Figure 1A; Supplementary Table S1). GSEA of the genes ranked by the ratios of transcripts from control and SMYD3-depleted cells identified significant enrichments of nine gene sets with *P*-value of <0.01 and FDR <0.05 (Supplementary Table S5). Representative GSEA-scoring plots and their corresponding heat maps indicating the enrichments of proliferation/invasion-related genes are shown (Figure 1B). IPA functional analysis of the repressed genes revealed that many of the genes down-regulated upon SMYD3 knockdown encode cell death/survival and cell proliferation/growth regulators (Supplementary Table S6), including those known to be key components of cancer initiation and progression. Our transcript profiling also showed that 84 genes were activated when SMYD3 was depleted. However, growth-stimulatory and proliferative genes appeared preferentially down-regulated under SMYD3 knockdown conditions (Supplementary Tables S6 and S7), suggesting that SMYD3-mediated activation of target genes is more functional in cancer cells. These genes also tend to exhibit higher ratios of expression changes, which is in agreement with the primarily activating function expected for SMYD3 during cancer development (2,10,12,13). As an experiment to confirm the microarray results, qRT-PCR analysis of 17 putative target genes in HCT116 and three other cancer cell lines (CaCO2, J82 and T24) showed that SMYD3 knockdown caused 40–70% decreases in their mRNA levels (Supplementary Figures S2 and S4). To further validate our microarray results, we checked the rescue potential of ectopic SMYD3 (Supplementary Figure S3A). The expression of SMYD3 wild-type in SMYD3-depleted cells fully restored the transcription of target genes, whereas SMYD3 enzymatic dead mutant (F183A) was much less efficient in restoring their transcription rates (Figure 1C).

Since SMYD3 functions as a transcription regulator by binding to a specific DNA sequence (5′-CCCTCC-3′) in its target genes, we searched for this SMYD3 binding element around the transcription start sites (TSSs) of up- and down-regulated genes. We found that 176 (67%) out of 264 down-regulated genes and 49 (58%) out of 84 up-regulated genes in SMYD3-depleted cells carry the potential SMYD3 binding site (Supplementary Table S8). Thus, it is likely that the site specific binding of SMYD3 to its consensus site underlie the ability of SMYD3 to regulate these target genes. To explore this possibility, we tested whether SMYD3 was bound to the four selected target genes (*FNBP1, MFGE8, PDLIM7* and *WNT3A*) which were repressed after SMYD3 knockdown and contain putative SMYD3 binding elements within 300 bp upstream of the TSSs (Supplementary Figures S5A and S5B). The selected target genes are known to facilitate cell proliferation via integrin/ERK1/2 signaling (31) or ERK-mediated Op18/stathmin signaling (32) and cell invasion via invadopodia formation (33) or epithelial-mesenchymal transition (34). As expected, ChIP and quantitative PCR (ChIP-qPCR) assays showed high levels of SMYD3 occupancy at the SMYD3 binding elements in mock-depleted bladder (J82 and T24)/colon (HCT116 and CaCO2) cancer cells, but SMYD3 levels were markedly decreased after SMYD3 knockdown (Supplementary Figure S5C). On the contrary, ChIP analysis showed no enrichments of SMYD3 at the *KRT81* gene whose expression was not affected by SMYD3 knockdown in our microarray analysis. The presence of a putative SMYD3 cognate element in this gene indicates that SMYD3 is incapable of binding to all genes containing its cognate element. To investigate the mechanisms by which SMYD3 depletion leads to inactivation of target genes, we also checked H3K4me3 on target genes in mock-depleted and SMYD3-depleted cells. High levels of H3K4me3 were detected around the SMYD3 binding elements of target genes, but such enrichment was significantly reduced upon SMYD3 depletion (Supplementary Figure S5D). These results imply that SMYD3 is important for establishing the active H3K4me3 mark on proliferation/invasion-stimulatory genes in cancer cells.

In order to gain further insight into how SMYD3 regulates its target genes, SMYD3 occupancy was probed for the six different regions of the *FNBP1* and *MFGE8* genes by ChIP-qPCR, as depicted in Figure 1D and Supplementary Figure S3B. In mock-depleted control cells, SMYD3 signals were high at the proximal promoter region containing a putative SMYD3 binding element (region C) and proximal coding region (region D), but low SMYD3 signals were detected at four other regions (regions A, B, E and F) (Figure 1D and Supplementary Figure S3B). It has been demonstrated that SMYD3 participates in the early steps of transcriptional elongation and facilitates the transition from transcription initiation to early elongation (8). This notion is supported by the fact that the levels of SMYD3 occupancy are higher at the proximal coding region (position D) than at promoter region (position C). Moreover, SMYD3 showed a pattern of accumulation indistinguishable from that of H3K4me3, strongly suggesting a major role for SMYD3 in mediating higher levels of H3K4me3 in these target genes (Figure 1D and Supplementary Figure S3B). Indeed we found that SMYD3 knockdown reduced levels of SMYD3 in the *FNBP1* and *MFGE8* genes, and such changes diminished H3K4me3 levels (Figure 1D and Supplementary Figure S3B). These results were further validated by rescue experiments demonstrating that ectopic expression of SMYD3 wild-type (wt), but not SMYD3 enzymatic dead mutant (F183A), largely overrides H3K4me3 defects caused by SMYD3 knockdown. MTT and cell invasion assays also showed that SMYD3 depletion decreased the proliferation and invasion of HCT116 cancer cells and that the expression of SMYD3 wild-type, but not SMYD3 F183A mutant, restored cell proliferation and invasion rates (Figure 1E and F).

### SMYD3 interacts directly with PC4

**Figure 2.**
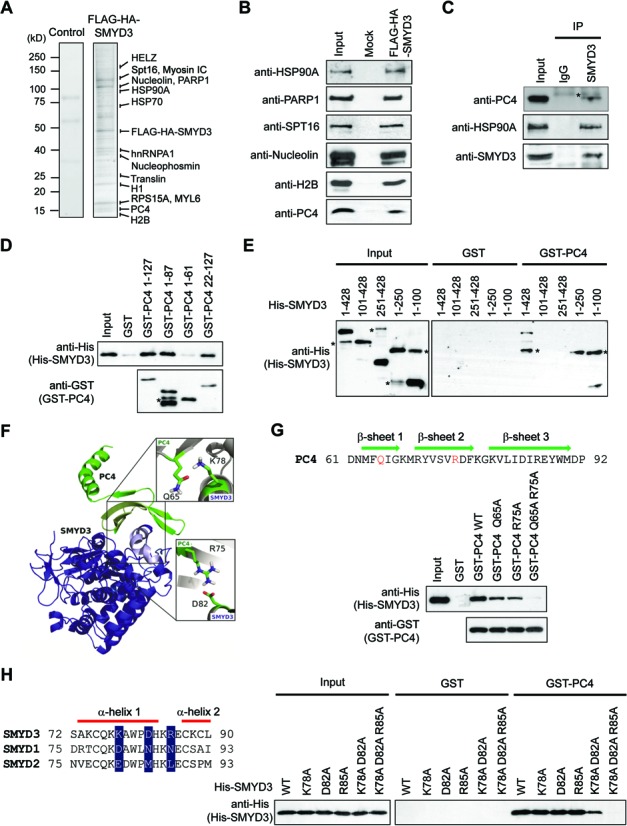
PC4 is an interaction partner of SMYD3. (**A**) FLAG-HA-tagged SMYD3 was expressed in SMYD3-depleted HCT116 colon cancer cells and sequentially immunoprecipitated using anti-FLAG and anti-HA antibodies. The co-purified proteins were separated by 4–20% gradient SDS-PAGE and identified by liquid chromatography-tandem mass spectrometry (LC-MS/MS). The positions of the molecular mass markers are indicated on the left. (**B**) The purified SMYD3 sample shown in (**A**) was resolved in 4–20% gradient SDS-PAGE and analyzed by immunoblotting with the indicated antibodies. (**C**) Whole cell lysates were prepared from HCT116 cells, immunoprecipitated with anti-SMYD3 antibody or control IgG, and analyzed by immunoblotting with anti-PC4, anti-HSP90A and anti-SMYD3 antibodies. Input represents 20% of the cell extracts used in immunoprecipitation. Asterisk indicates non-specific bands. (**D**) GST alone or GST-PC4, immobilized on glutathione-Sepharose beads, was incubated with full length His-SMYD3 or its deletion mutants. After extensive washing with washing buffer, bound SMYD3 proteins were fractionated by 10% SDS-PAGE, and immunoblotted with anti-His antibody. Five percent of the input proteins were examined by immunoblotting. (**E**) His-tagged SMYD3 was incubated with immobilized GST or the indicated GST-H1.2 fusions, and bound SMYD3 proteins were visualized by immunoblotting with anti-His antibody. Input represents 5% of SMYD3 protein used in the binding reactions. Asterisks indicate non-specific bands. (**F**) The model of the SMYD3–PC4 complex was built on the basis of crystal structures of SMYD3 (4) and PC4 (19,20) using the molecular modeling program Insight II (Molecular Simulations Inc.). Residues located at the interface are indicated. SMYD3 is in blue and PC4 is in green. (**G**) Pull-down experiments were performed as described in (**E**), but using GST-PC4 1–127 carrying the indicated point mutations. The three short parallel β-sheets are indicated above the sequence. (**H**) GST pull-down assays were conducted as in (**E**), but using His-SMYD3 1–428 harboring the indicated point mutants. Alignment of the amino-acid stretch encompassing the PC4 binding region of SMYD3 relative to SMYD1 and SMYD2 is shown on the left for comparison. The α-helical structural elements are indicated above the sequence.

Overall, our microarray and ChIP data establish the role for SMYD3 in regulating particular sets of genes in bladder and colon cancer cells. However, it is not clear whether the observed effects of SMYD3 reflect its physical interaction with other factors. To check this possibility, we generated HCT116 colon cancer cell lines stably expressing SMYD3 fused to FLAG and HA epitope tags. After preparing nuclear extracts from cultured cells, ectopic SMYD3 and its associated partners were purified from nuclear extracts through sequential immunoprecipitations with FLAG and HA antibodies under stringent conditions (300 mM KCl and 0.1% NP40). The proteins co-purified with SMYD3 were resolved by SDS-PAGE, and major protein bands were excised and subjected to tandem mass spectrometric analysis in conjunction with a sequence database search. As summarized in Supplementary Table S9, we detected the stable association of 15 proteins with ectopic SMYD3 in this analysis. As predicted from recent studies, we identified HELZ and HSP90A that have been shown to interact with SMYD3 (2,35). Interestingly, our analysis also revealed 13 proteins that have never been described in association with SMYD3 (Figure 2A; Supplementary Table S9). The mass spectrometry data were validated by immunoblot analysis using available antibodies (Figure 2B). A noteworthy observation emerged from our purification was a substantial interaction of SMYD3 with PC4, which is a transcription factor possessing the ability to co-activate RNA polymerase II-mediated transcription. PC4 has been reported to be overexpressed in several types of cancer and contribute to tumorigenesis via transcriptional activation of other genes involved in cancer pathogenesis (24,25,36). PC4 has also been shown to play a role in the regulation of chromatin transcription through its influence on histone modifications (23,37). Thus the fact that PC4 stably associates with SMYD3 in our purification prompted us to study the possible influences of PC4 on SMYD3-mediated transactivation and cancer cell proliferation/invasion.

To gain support for the binding results described above, we first immunoprecipitated HCT116 cell lysates with anti-SMYD3 antibody and checked the coimmunoprecipitation of endogenous PC4. In addition to SMYD3, we could confirm the presence of PC4 in our immunoprecipitates (Figure 2C). We then conducted in vitro pull-down assays using bacterially produced His-tagged SMYD3 and GST-fused PC4. As shown in Figure 2D, GST-PC4 efficiently interacted with His-SMYD3, whereas GST alone did not. In similar binding experiments with truncated versions of PC4, SMYD3 interacted with two PC4 deletion mutants lacking the first 21 or last 40 residues (PC4 22–127 and PC4 1–87), but not with another PC4 mutant lacking the last 66 residues (PC4 1–61), indicating that amino acid residues 62–87 play a major role in PC4 binding to SMYD3. In mapping PC4-interacting region of SMYD3, the binding of the N-terminal domains (residues 1–100 and 1–250) was readily detectable, but the two C-terminal domains (residues 101–428 and 251–428) showed no interaction with PC4 (Figure 2E). In additional binding assays, SMYD3 deletion mutants lacking residues 1–44 and 1–74 retained their affinity for PC4, while no apparent interaction was observed with two SMYD3 mutants lacking residues 75–87 and 75–100 (Supplementary Figure S6). These results reinforce the conclusion that the primary PC4-binding capacity of SMYD3 resides between residues 75 and 87.

We next attempted to identify amino acid residues critical for the SMYD3–PC4 interaction. Circular dichroism spectroscopy of SMYD3, PC4 and the SMYD3–PC4 complex showed that all proteins were folded with the complex representing the superposition of the individual structures (Supplementary Figure S7). To facilitate the search for residues in the above-outlined interaction interface, we created a structural model of the SMYD3–PC4 complex using the crystal structures of SMYD3 (4) and PC4 (19,20). Our model highlighted the N-terminal helix of SMYD3 and the central β-sheet of PC4 to be important in their interaction (Figure 2F). Specifically, K78 and D82 of SMYD3 appeared positioned to make contacts with Q65 and R75 of PC4, respectively (Figure 2F). Individual mutation of Q65 and R75 to alanine had some effect on the PC4 interaction with SMYD3 (Figure 2G, low panel). However, an essentially complete abrogation of the PC4–SMYD3 interaction required the alanine substitution of both Q65 and R75. In similar binding assays using SMYD3 mutants, the mutation of K78 and D82 individually did not affect SMYD3 interaction with PC4, and mutation of both residues only minimally decreased PC4 binding activity of SMYD3 (Figure 2H, right panel). This result indicated that additional SMYD3 residues were important for the interaction with PC4. Among the SMYD family, R85 is, aside K78 and D82, unique to the PC4 binding domain of SMYD3 (Figure 2H). We found that simultaneous alanine substitutions of K78, D82 and R85 significantly incapacitated SMYD3 from interacting with PC4, whereas R85-mutated SMYD3 had a binding affinity comparable to wild-type SMYD3 (Figure 2H). These results clearly establish SMYD3–PC4 interaction centered on the SMYD3 N-terminal α-helix and PC4 central β-sheet, suggesting that PC4 can directly modulate SMYD3 transactivation.

### SMYD3 and PC4 cooperate to drive gene expression program in cancer cells

**Figure 3.**
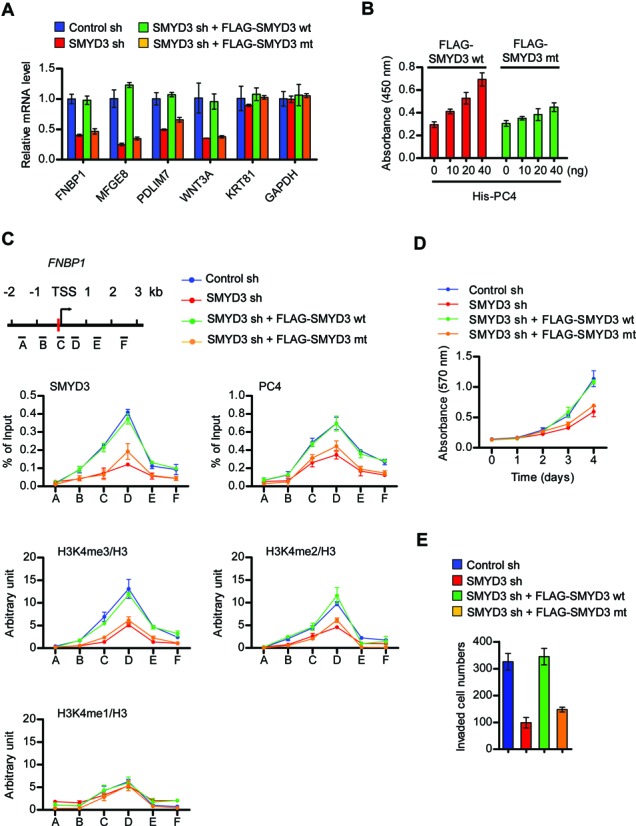
SMYD3 is required for PC4 localization and function at target genes. (**A**) SMYD3-depleted HCT116 cells were infected with SMYD3 wild-type (wt) or K78/D82/R85 mutant (mt) as in Supplementary Figure S7A, and the levels of target gene expression were measured by qRT-PCR. (**B**) FLAG-tagged versions of SMYD3 wt and SMYD3 mt were stably bound to biotin-conjugated SMYD3 binding sites attached to avidin-coated 96 well plates. After incubating wells with increasing concentrations of His-tagged PC4 wt, changes in SMYD3 affinity to its binding sites were quantitatively analyzed by sequential incubations with anti-FLAG antibody, a horseradish peroxidase conjugated secondary antibody, and a colorimetric peroxidase substrate. Absorbance was read at 450 nm on a microplate reader. (**C**) ChIP-qPCR assays of the *FNBP1* gene were carried out using indicated antibodies in control or SMYD3-depleted HCT116 cells complemented with SMYD3 wt or SMYD3 mt. ChIP-enriched DNA was quantified by qPCR using the primers indicated in the scheme at the top. (**D**) SMYD3-depleted HCT116 cells were complemented with SMYD3 wt or SMYD3 mt, and MTT proliferation assays were carried out over a period of 4 days. (**E**) Matrigel cell invasion assays were performed with SMYD3-depleted HCT116 cells expressing SMYD3 wt or SMYD3 mt.

The observed interaction between SMYD3 and PC4 raised the possibility that SMYD3 functionally cooperates with PC4 to establish the active state of target genes in bladder and colon cancer cells. Congruent with these ideas, when the genes that were down-regulated in SMYD3-depleted cells were analyzed by qRT-PCR, their transcription levels were completely recovered after the ectopic expression of SMYD3 (Figure 3A and Supplementary Figure S8A). However, the expression of SMYD3 K78/D82/R85 mutant, which is unable to interact with PC4, failed to show any recovery effect on target gene transcription (Figure 3A). Having demonstrated a functional interaction between SMYD3 and PC4, we then asked whether the observed function of PC4 in SMYD3 transactivation reflects its effects on SMYD3 binding to target DNA sequences. To this end, we immobilized biotinylated SMYD3 cognate DNA elements on streptavidin-coated microtiter plates and carried out SMYD3 binding assays in the presence of PC4. In our color reaction-based measurements of SMYD3 binding affinity, more efficient association of SMYD3 with its binding elements was seen if SMYD3 was added with increasing concentration of PC4, while only minor effects of PC4 were observed when SMYD3 mutant was used (Figure 3B).

To elucidate the significance of PC4 with respect to SMYD3 transactivation, we next investigated PC4 localization at the *FNBP1* and *MFGE8* genes by ChIP assays employing cross-linked chromatin isolated from control and SMYD3-depeleted cancer cells. Immunoblotting confirmed that SMYD3 knockdown had no effect on the expression levels of PC4 in HCT116 cells (Supplementary Figure S8A). The distribution patterns of PC4 across the *FNBP1* and *MFGE8* genes were similar to those of SMYD3 and H3K4me3, with a peak around the proximal promoter and coding regions (regions C and D) and a gradual decrease over the distal promoter and coding regions (regions A, B, E and F) (Figure 3C and Supplementary Figure S8B). By comparison, SMYD3 depletion reduced the levels of PC4 at target genes, strongly indicating that SMYD3 is indispensable for the initial recruitment of PC4 to target genes. Although beyond the scope of the present study, higher PC4 occupancy at the proximal coding region (position D) is consistent with accumulating evidence suggesting that PC4 stimulates transcription beyond the initiation stage (17,19,22,38). The requirement of SMYD3 in PC4 recruitment was further supported by the fact that expressing SMYD3 wild-type, but not SMYD3 K78/D82/R85 mutant which is deficient in PC4 binding, in SMYD3-depleted cells resulted in a substantial increase in the levels of PC4 at target genes. Given that SMYD3 promotes cell proliferation and invasion, we also examined whether SMYD3 capacity to interact with PC4 is necessary for the observed effects. Unlike SMYD3 wild-type, PC4 binding-deficient SMYD3 mutant failed to recover the proliferation and invasiveness of SMYD3-depleted cancer cells (Figure 3D and E), thus demonstrating that SMYD3-mediated recruitment of PC4 is critical for SMYD3 function at genes involved in cell proliferation and invasion.

**Figure 4.**
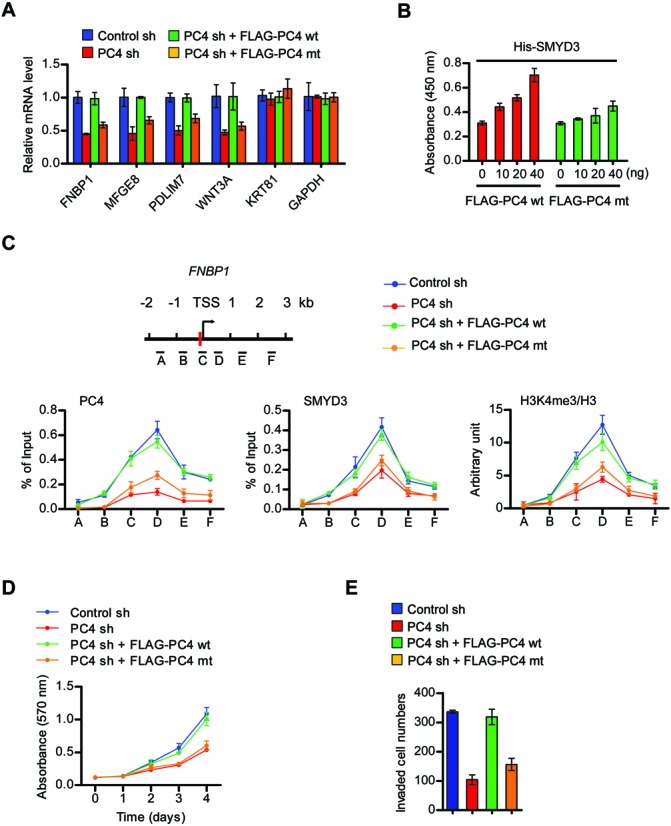
PC4 regulates SMYD3 occupancy and function at target genes. (**A**) Total RNAs from control or PC4-depleted HCT116 cells complemented with PC4 wt or PC4 mt were subjected to qRT-PCR analysis to assess target gene expression levels. (**B**) Quantitative binding assays were performed as described in Figure 3B, but using His-SMYD3 wt together with FLAG-tagged PC4 wt or PC4 mt. (**C**) ChIP-qPCR assays were performed in control or PC4-depleted HCT116 cells complemented with PC4 wt or PC4 mt. The relative position of the qPCR primers is indicated at the top. (**D**) MTT proliferation assays were performed with PC4-depleted HCT116 cells complemented with PC4 wt or PC4 mt over a period of 4 days. (**E**) Invasive potential of PC4-depleted HCT116 cells complemented with PC4 wt or PC4 mt was monitored by Matrigel invasion assay.

To further investigate the impact of PC4 on SMYD3function, we depleted PC4 and evaluated its effects on target gene expression. As confirmed by immunoblot, the transfection of PC4-targeted shRNA resulted in an almost complete disappearance of PC4 without affecting SMYD3 expression in HCT116 cells (Supplementary Figure S8C). PC4 knockdown generated a distinct repression of SMYD3 target genes, and PC4 wild-type, but not SMYD3-bindining deficient PC4 mutant, could rescue the expression of target genes (Figure 4A and Supplementary Figure S8C). In our mechanistic studies addressing how PC4 contributes to transactive function of SMYD3 at target genes, free SMYD3 exhibited circular dichroism spectra almost identical to those of PC4-bound SMYD3 (Supplementary Figure S7), thus ruling out the possibility that PC4 alters SMYD3 structure more favorable for its DNA binding. Another possibility is that PC4 interacts with SMYD3 MYND domain bound to its target sites, and stabilizes the SMYD3–DNA interface without altering overall structures. Consistent with this idea, our binding assays employing SMYD3 cognate DNA elements immobilized on streptavidin-coated microtiter plates demonstrated that SMYD3 binds to its target sequence more strongly in the presence of PC4 wild-type compared to PC4 mutant (Figure 4B). ChIP analyses to elucidate the nature of PC4 recruitment patterns further revealed a perfect correlation of PC4 localization with SMYD3 occupancy and H3K4me3 enrichment on the *FNBP1* and *MFGE8* genes (Figure 4C and Supplementary Figure S8D). PC4 occupancy of the target genes was reduced after knockdown or Q65/R75 mutation of PC4, but such changes also diminished the levels of SMYD3 and H3K4me3. The rescue of PC4 knockdown resulted in a return of SMYD3-mediated H3K4me3, demonstrating the reversibility of this modification process. As a control, SMYD3 knockdown and rescue had no effect on PC4 localization and transcription activity at the *SMUG1* gene which is a known PC4 target gene and is not affected by SMYD3 knockdown in our microarray studies (Supplementary Figure S9). The importance of PC4 with respect to SMYD3 function was further confirmed by the finding that wild-type PC4 fully restored the proliferation rates of the cancer cells depleted of PC4, whereas SMYD3 binding-deficient PC4 was much less efficient in restoring the proliferation rates (Figure 4D). Essentially identical results were obtained from invasion assays (Figure 4E). Thus, the expression of wild-type PC4, but not SMYD3 binding-deficient PC4, rescued cell invasion rates.

### Artificial tethering of PC4-binding deficient SMYD3 to target genes can generate

#### H3K4me3 and transactivation

**Figure 5.**
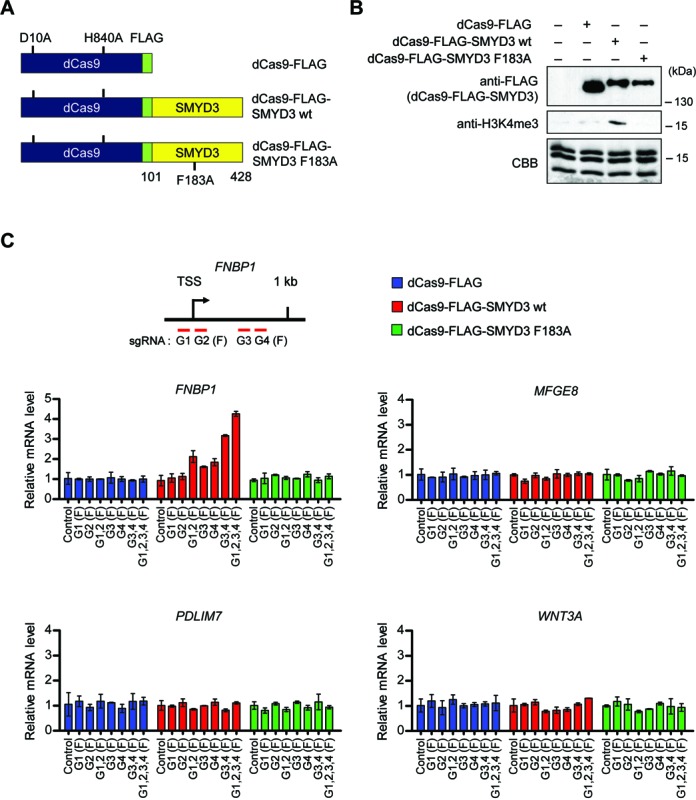
dCas9-SMYD3 fusion activates transcription of target genes. (**A**) Schematic diagram of the CMV-based constructs driving expression of dCas9-FLAG, dCas9-FLAG-SMYD3 wild-type (wt) and dCas9-FLAG-SMYD3 enzymatic dead mutant (F183A). (**B**) Nuclear extracts were prepared from 293 cells transfected with plasmids expressing dCas9-FLAG, dCas9-FLAG-SMYD3 wt or dCas9-FLAG-SMYD3 F183A, and were subjected to immunoprecipitation using anti-FLAG antibody. Immunoprecipitates were then assayed for H3K4 specific HMT activity using histone octamers as substrates. The positions of the molecular mass markers are indicated on the right. (**C**) Approximate locations of sgRNA pairs targeting the *FNBP1* locus are shown on the top. The sgRNA sequences are listed in Supplementary Table S2. 293 cells were transfected with the indicated dCas9 and sgRNA expression constructs for 48 h, and total RNA was isolated and analyzed by qRT-PCR using primers specific for the *FNBP1, MFGE8, PDLIM7* and *WNT3A* genes.

The results of the above ChIP and knockdown experiments argue persuasively that PC4 is indispensable for the stable recruitment of SMYD3 to target genes. However, these studies do not discount the possibility that PC4 could contribute to SMYD3 transactivation via some other mechanisms. If PC4 exerts its cooperative effects mainly by enhancing SMYD3 binding to its cognate elements, then we can predict that artificially directing a SMYD3 mutant incapable of interacting with PC4 and binding to target genes is sufficient to establish an active state of transcription. To examine this possibility, we chose to adapt CRISPR system for targeting SMYD3-mediated H3K4me3 to specific genes. We created genes encoding catalytically dead Cas9-FLAG (dCas9-FLAG) and dCas9-FLAG fused to N-terminal truncated SMYD3 (residues 101–428) wild-type (wt) or enzymatic dead mutant (F183A), and constructed their expression plasmids using pIRES mammalian expression vector containing the CMV promoter (Figure 5A). When dCas9 fusion proteins were immunoprecipitated from transfected cells and analyzed by HMT assays, dCAS-FLAG-SMYD3 wild-type, but not its mutant counterpart (F183A), methylated H3 in histone octamers (Figure 5B). These results indicate that the N-terminal domain (amino acids 1–100) is not necessary for SMYD3 enzymatic activity toward histone H3 in the Cas9 system. We also generated plasmids expressing four different small guide RNAs (sgRNAs) targeted to the proximal promoter (G1 and G2) and coding (G3 and G4) regions of the *FNBP1* and *MFGE8* genes under the control of U6 promoter (Figure 5C and Supplementary Figure S10, upper left panel). We then transfected dCas9 fusion proteins into 293 cells that express SMYD3 at low to undetectable levels (2) together with one to four separate sgRNA expression constructs targeting either the *FNBP1* or *MFGE8* gene. It is noteworthy that in the absence of N-terminal domain (residues 1–100), SMYD3 can neither interact with PC4 (Figure 2E) nor bind to its cognate DNA sequence in target genes (4). Thus, although *FNBP1* and *MFGE8* genes contain SMYD3 cognate elements, dCas9-SMYD3 has no specific DNA binding ability, and can activate gene expression, only when guided to target genes by sgRNAs. Also, the use of the N-terminally truncated SMYD3 in dCAS9-SMYD3 fusion constructs excludes the possible effects of endogenous PC4 on our CRISPR experiments.

As shown in Figure 5C and Supplementary Figure S10 (middle left panels), individual transfection of G3 and G4 sgRNA constructs into cells expressing dCas9-FLAG-SMYD3 resulted in a detectable enhancement of *FNBP1* and *MFGE8* mRNA levels (G3 and G4), whereas G1 or G2 sgRNA transfection displayed no obvious changes in transcription (G1 and G2). Also, targeting dCas9-FLAG-SMYD3 to the proximal promoter region by G1 and G2 sgRNA pair or to proximal coding region by G3 and G4 sgRNA pair activated the target genes by ∼2–3 fold (G1,2 and G3,4). To further support these results, expression of all four sgRNAs together with dCas9-FLAG-SMYD3 generated even higher elevation in *FNBP1* and *MFGE8* expression (G1,2,3,4). In parallel assays, dCas9-FLAG-SMYD3 enzymatic dead mutant (F183A) failed to show any change in transcription (dCas9-FLAG-SMYD3 F183A), clearly indicating the specificity and essential role of SMYD3 HMT activity in SMYD3 function on target genes. The targeting specificity of sgRNA-guided dCas9-FLAG-SMYD3 was further confirmed by the observation that sgRNAs targeting the *FNBP1* and *MFGE8* genes caused no silencing effects on *MFGE8* and *FNBP1* gene activation, respectively (Figure 5C and Supplementary Figure S10, middle right panel). Also of note, transfecting dCas9-FLAG-SMYD3 into cells expressing sgRNAs targeting *FNBP1*/*MFGE8* genes had no effect on two other SMYD3 target genes *PDLIM7* and *WNT3A* (Figure 5C and Supplementary Figure S10, bottom panel).

**Figure 6.**
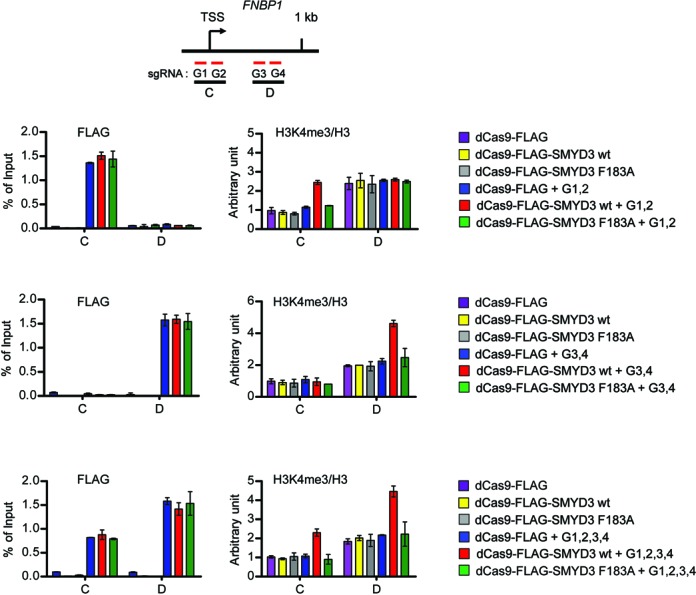
CRISPR/dCas9 system guides SMYD3 to target genes and generates H3K4me3. 293 cells were transfected with dCas9 and sgRNA expression constructs as in Figure 5, and the levels of dCas9 fusions and H3K4me3 at the proximal promoter and coding regions (regions C and D) of the *FNBP1* gene were assessed by ChIP. Precipitation efficiencies were determined for the two regions by qPCR with primers listed in Supplementary Table S4.

Based on the transcription results described above, we then tested the ability of dCas9-FLAG-SMYD3 to bind to target sites and generate H3K4me3 by ChIP analysis. Two sets of primers were employed to detect dCas9-FLAG-SMYD3 and H3K4me3 in the proximal promoter and coding regions by qPCR (Figure 6 and Supplementary Figure S11, first/top panel). Cotransfection of dCas9-FLAG-SMYD3 wild type or enzymatic dead mutant (F183A) with G1 and G2 sgRNAs resulted in a specific accumulation of the dCas9 fusion proteins in the proximal promoter regions of the *FNBP1* and *MFGE8* genes, whereas the dCas9 fusion proteins were mainly localized in the proximal coding regions when transfected with G3 and G4 sgRNAs (Figure 6 and Supplementary Figure S11, second and third panels, FLAG). In exploring the alteration of H3K4me3, we found that H3K4me3 levels were increased in the proximal promoter regions, when dCas9-FLAG-SMYD3 was cotransfected with G1 and G2 sgRNAs, but not with G3 and G4 sgRNAs (second and third panels, H3K4me3/H3). Expectedly, parallel ChIP assays on the proximal coding regions repeatedly demonstrated efficient accumulation of H3K4me3 in cells transfected with dCas9-FLAG-SMYD3 and G3 and G4 sgRNAs (second and third panels, H3K4me3/H3). By simultaneously transfecting all four sgRNAs, we also found that dCas9-FLAG-SMYD3 is able to mediate H3K4me3 at both proximal promoter and coding regions (fourth/bottom panels). To further confirm the above results, the ChIP assays were repeated using cells transiently transfected with dCas9-FLAG-SMYD3 F183A; this catalytically inactive SMYD3 mutant failed to support H3K4me3 in all cases (Figure 6 and Supplementary Figure S11, second, third and fourth panels). These data argue strongly against the possibility that H3K4me3 is mediated by some other cellular proteins, and indicate that SMYD3 can accurately establish H3K4me3 and modulate transcription if stably recruited to the proximal promoter and coding regions of target genes. Also, these results overall suggest that the effect of PC4 on SMYD3 transactivation of growth-stimulatory genes is mainly related to its ability to enhance SMYD3 binding to its target DNA regions.

## DISCUSSION

It has been known for years that SMYD3 overexpression is related to transcriptional changes in cancer cells (2,11,12,39), but it is only recently that molecular studies have merged to elucidate the oncogenic actions of this chromatin regulator. Its canonical mechanism of action involves binding to cognate DNA sequence and recruiting other transcription factors which contribute to specific aspects of SMYD3-driven transcription. To search for SMYD3-regulated genes in cancer cells, the genome-wide regulatory potential of SMYD3 was analyzed by gene expression profiling of colon cancer cells. In agreement with recent reports (11,13,39), our microarray results indicated that the genes encoding cell proliferation and invasion regulators were down-regulated in response to SMYD3 knockdown, functionally linking SMYD3 to oncogenic gene expression. Earlier studies established the enzymatic activity of SMYD3 on H3K4 methylation is critical for SMYD3 transactivation function (2,10), but the observed function could occur independently of the ability of SMYD3 to mediate H3K4me3 (1,8). However, our examination of H3K4me3 at SMYD3 target genes revealed that SMYD3 knockdown has a major impact on H3K4me3 levels in the cancer cell lines employed in this study. Although these effects could be indirect, the striking correlation between the levels of SMYD3 and H3K4me3 at target genes suggests that SMYD3 is a prominent regulator of H3K4me3 in the cancer cell lines. Related, a recent report indicated that SMYD3 also methylates K5 on H4 tails in breast and liver cancer cell lines (40), suggesting a possible role of H4K5me3 in SMYD3-driven oncogenic processes. This observation might seem contradictory to our results; however, we provided compelling evidence that SMYD3 has a bona fide stimulation effect on H3K4me3 and that this activity is critical for target gene expression in our assay system. Thus, once H4K5me3-specific antibodies are available, it will be important to determine whether H4K5me3 may impact on chromatin function at the global level whereas H3K4me3 effects are rather focused on specific genes, as recently proposed (41).

The previous demonstration that SMYD3 participates in the recruitment of regulatory factors (e.g., BRD4, HELZ, HSP90A and p-TEFb) through protein-protein interaction makes it likely that other factors might be required for SMYD3-induced transactivation and tumorigenesis (2,8). Using affinity purification and mass spectrometric approaches, we purified proteins that stably associate with SMYD3 from colon cancer cells and identified PC4 as one of the associated factors. This suggests a possible cooperative function of PC4 in SMYD3 transcription network in cancer cells. Supporting this idea, our knockdown and rescue experiments provided a clear and convincing demonstration that SMYD3 transactivation of growth/invasion-stimulatory genes in cancer cells is dependent of PC4. Toward an understanding of the underlying mechanism, we demonstrated that there is a direct interaction between SMYD3 and PC4 which offers a mean for PC4 recruitment and stabilization at SMYD3 target genes. The molecular modeling and amino acid sequence alignment suggested that residues K78, D82 and R85 of SMYD3 and residues Q65 and R75 of PC4 are critical for SMYD3–PC4 interaction. In fact, the importance of these amino acids was confirmed by the finding that their mutations caused an impaired interaction of SMYD3 with PC4 and a corresponding loss of target gene transactivation. Thus, despite the fact that SMYD3 bears amino acid sequence similarity to other SMYD family members, several specific amino acids underlie the distinct structural and functional properties of SMYD3 in attracting PC4 to target genes and establishing active transcriptional states.

Another intriguing finding of our study is that the stable localization of SMYD3 at target genes is dependent upon PC4, which underscores a complex relationship between SMYD3 and PC4 in gene transcription. Further indicative of a PC4 role in enhancing SMYD3 occupancy at target genes is much lower levels of H3K4me3 in PC4-depleted cells, compared to control cells. The mechanistic basis for this reciprocal effect of PC4 on SMYD3 occupancy and function at target genes is not fully elucidated in this study. However, considering that SMYD3 binds to its cognate site through the MYND domain containing alpha helices and that PC4 beta sheet region interacts with SMYD3 MYND alpha helical motif, PC4-induced stabilization of SMYD3 alpha helical contact with target DNA sequence is likely to be part of the mechanism by which PC4 contributes to SMYD3-mediated transactivation. This finding also suggests that the presence of PC4 is critical for the stable accumulation of H3K4me3 at SMYD3 target genes, functionally linking PC4 to an active histone mark. Structural investigations of PC4-bound SMYD3 will provide information on the nature of SMYD3–PC4 interaction and how PC4 triggers a stable retention of SMYD3 at target genes. Also indicative of an apparent role of SMYD3 in early transcriptional elongation, our ChIP analysis detected higher amounts of SMYD3-mediated H3K4me3 at the proximal coding region than promoter region of target genes. Our results agree with those of previous work (8), which found that SMYD3 directly interacts with RNA polymerase II and facilitates the recruitment of transcription elongation factors. Thus it is tempting to speculate that SMYD3 moves along with RNA polymerase II during the transition step from initiation to elongation and mediates H3K4me3. These results also suggest that PC4 plays a role in the transition from transcription initiation to elongation in collaboration with SMYD3. Accordingly, we observed that PC4 is constitutively associated within the target genes in a manner overlapping with the distribution patterns of SMYD3 and H3K4me3. In fact, this is consistent with recent reports showing that PC4 actively orchestrates with other transcription regulators and chromatin remodelers during the early elongation steps (15,21). Understanding of the precise contributions of SMYD3 and PC4 to the organization of transcription programs downstream of TSSs awaits further experimentation.

Notably, and consistent with the primary mechanism of action of PC4 in SMYD3 transactivation, our CRIPSR experiments demonstrated that simultaneous transfection of dCas9-FLAG-SMYD3 (residues 101–428) and sgRNA sets targeting the proximal promoter and coding regions was sufficient to boost the transcription of two SMYD3 target genes *FNBP1* and *MFGE8* in 293 cells. Individual sgRNA could not effectively activate the corresponding target genes, whereas a mixture of two or four sgRNAs efficiently generated transcriptional activation. Currently, we do not have mechanistic explanation about this phenomenon, but we think that multiple sgRNAs increase the likelihood of dCas9-FLAG-SMYD3 localization at a sufficiently high level to promote transcription. In additional support of the physiological relevance of the transcription results, our ChIP analyses showed that transcriptional activation of endogenous target genes by dCas9-SMYD3 coincides with H3K4me3. Perhaps more important are observations that there is a strong correlation between the level of dCas9-SMYD3-mediated H3K4me3 and the level of dCas9-SMYD3-induced transcriptional activation and that dCas9-SMYD3 enzymatic dead mutant is unable to generate productive transcription. Furthermore, our observation that combined application of sgRNAs targeting the proximal promoter and coding regions generates a synergistic effect is supportive of the idea that SMYD3 acts to regulate both initiation and elongation events. In relation to the use of N-terminal truncated version of SMYD3 in our CRIPSR experiments, a previous study suggested that the N-terminal domain of SMYD3 is important for SMYD3 HMT activity and histone substrate specificity (42). However, our analysis of dCas9-SMYD3 revealed that N-terminal deleted SMYD3 is able to mediate H3K4me3 in our CRISPR experiments. This result is consistent with the idea, supported by studies of a naturally occurring N-terminal deletion mutant of SMYD3 (43), that the N-terminal domain is dispensable for SMYD3 enzymatic activity toward histone H3. It is our view that the N-terminal domain plays a role in modulating the DNA binding affinity and specificity of SMYD3.

In summary, we have demonstrated that SMYD3 promotes cell proliferation and invasion by mediating H3K4me3 and PC4 recruitment at the proximal promoter and coding regions of target genes, thereby positively influencing their expression in bladder and colon cancer cells. Dynamic regulation of SMYD3-mediated H3K4me3 by PC4 also contributes to productive transcription of SMYD3 responsive genes in cancer cells. Thus, targeting therapeutic intervention to cooperative activity of SMYD3 and PC4 could provide effective strategy for cancer treatment.

## Supplementary Material

SUPPLEMENTARY DATA
