# Supplementary material for: Cooperation between SMYD3 and PC4 drives a distinct transcriptional program in cancer cells
Source: Nucleic Acids Res. 2015 Oct 10;43(18):8868–83. doi: 10.1093/nar/gkv874 (PMC4605318; doi:10.1093/nar/gkv874)
Supplement: SUPPLEMENTARY DATA [file supp_43_18_8868__index.html]

Cooperation between SMYD3 and PC4 drives a distinct transcriptional program in cancer cells — SUPPLEMENTARY DATA 

# Cooperation between SMYD3 and PC4 drives a distinct transcriptional program in cancer cells

## SUPPLEMENTARY DATA

- SUPPLEMENTARY DATA
- SUPPLEMENTARY DATA
- SUPPLEMENTARY DATA
- SUPPLEMENTARY DATA
