# Supplementary material for: Cooperation between SMYD3 and PC4 drives a distinct transcriptional program in cancer cells
Source: Nucleic Acids Res. 2015 Oct 10;43(18):8868–83. doi: 10.1093/nar/gkv874 (PMC4605318; doi:10.1093/nar/gkv874)
Supplement: SUPPLEMENTARY DATA [file supp_gkv874_nar-01044-x-2015-File008.pdf]

Supplemental Information

Cooperation between SMYD3 and PC4 drives a distinct transcriptional program in cancer cells

Jin-Man Kim, Kyunghwan Kim, Thomas Schmidt, Vasu Punj, Haley Tucker, Judd C. Rice, Tobias S. Ulmer and Woojin An

Supplementary Figure Legends

Figure S1. Effects of SMYD3 knockdown on proliferation and invasion of bladder and colon cancer cells.
(**A**) Whole cell extracts were prepared from colon normal (CDC-18Co) and cancer (HCT116, CaCO2, and HT29) cells, bladder normal (UROtsa) and cancer (J82, T24 and RT4) cells, breast normal (MCF-10-2A) and cancer (MCF7 and MDA-MB-231) cells, and prostate normal (MLC) and cancer (LNCaP and DU145) cells. Equal amounts of extracts were analyzed by immunoblotting with anti-SMYD3 and anti-Actin antibodies. (**B**) HCT116, CaCO2, J82 and T24 cancer cells were transfected with lentiviruses expressing control shRNA (Control sh) or SMYD3-specific shRNA (SMYD3 sh), and knockdown efficiency was determined by immunoblotting. (**C**) HCT116, CaCO2, J82 and T24 cancer cells were transfected with control shRNA or SMYD3 shRNA, and their proliferation rates were determined by MTT colorimetric assays over a period of 4 days. Results represent the means of three independent experiments ± SD. (**D**) Control or SMYD3-depleted HCT116, CaCO2, J82 and T24 cancer cells were detached and seeded onto the upper chamber coated with Matrigel, and then allowed to invade toward 10% FBS in the lower chamber. The graph depicts the average number of invaded cells per four fields. Results represent the means ± SD of three independent experiments.

Figure S2. Validation of microarray data.
Microarray data were validated by qRT-PCR of seventeen genes whose expression was decreased upon SMYD3 knockdown and which are related to cell growth and invasion, and two unaffected genes. Primer sequences are listed in Table S3. The values are expressed as fold changes from the mRNA levels in control cells after normalization with *ACTB*. Data represent the means ± SD of three independent experiments.

Figure S3. Target gene occupancy of SMYD3 and H3K4me3.
(**A**) SMYD3-depleted HCT116 colon cancer cells were infected with lentiviruses expressing RNAi-resistant FLAG-SMYD3 wild-type (wt) or enzymatic dead mutant (F183A), and whole cell extracts were analyzed by immunoblotting with the indicated antibodies. (**B**) ChIP-qPCR experiments were performed as in Figure 1D, but using primers specific for the *MFGE8* gene. The vertical red bar represents the putative SMYD3 binding site.

Figure S4. Effects of SMYD3 knockdown on target gene expression.
Expression levels of the genes down-regulated (*FNBP1*, *MFGE8*, *PDLIM7*, and *WNT3A*) or unaffected (*KRT81* and *GAPDH*) were analyzed by qRT-PCR on total RNA isolated from SMYD3-depleted CaCO2, J82 and T24 cancer cells.

Figure S5. Effects of SMYD3 knockdown on H3K4me3.
(**A**) Potential SMYD3 binding sites in target gene promoters are indicated in red. The nucleotides in blue indicate primer sequences used for ChIP-qPCR. (**B**) Diagram depicting the positions of the PCR amplicons used for ChIP assays. The numbers represent the start and end positions of amplicons relative to the transcription start site (TSS). The putative SMYD3 binding sites are shown as vertical red bars. (**C** and **D**) ChIP experiments determining SMYD3 localization (**C**) and H3K4me3 enrichment (**D**) at the four SMYD3 target genes (*FNBP1*, *MFGE8*, *PDLIM7*,

WNT3A) and two control genes (*KRT81* and *GAPDH*) in SMYD3-depleted HCT116, CaCO2, J82 and T24 cancer cells using primers depicted in (**B**).

Figure S6. Interaction of SMYD3 N-terminal deletion mutants with PC4.
GST alone or GST-PC4, immobilized on glutathione Sepharose beads, was incubated with His-tagged SMYD3 deletion mutants. After washing with washing buffer, the bound SMYD3 proteins were immunoblotted with His antibody. Of the input proteins, 5% were examined by immunoblotting.

Figure S7. Circular dichroism spectra of SMYD3 and PC4.
Spectra of SMYD3 (black) and PC4 (red) are shown. In addition, the spectra of the SMYD3-PC4 complex is depicted in green, which matches the sum of the individual SMYD3 and PC4 spectra, shown in blue. Spectra were recorded at protein concentrations of 5 μM in 5 mM K_2HPO_4/KH_2PO_4, pH 7.4, 25 mM KCl at 25 °C using a JASCO J-810 spectropolarimeter. The observed ellipticity in millidegrees, θ, was converted into the mean residue ellipticity, θ_{MRW}.

Figure S8. Interdependent localization of SMYD3 and PC4 at target genes.
(**A**) SMYD3-depleted HCT116 colon cancer cells were infected with lentiviruses expressing FLAG-tagged SMYD3 wild-type (wt) or K78/D82/R85 mutant (mt) lacking interaction with PC4, and cell lysates were prepared and analyzed by immunoblotting with anti-SMYD3, anti-FLAG, anti-PC4, and anti-Actin antibodies, as indicated. (**B**) ChIP-qPCR experiments were performed as in Figure 3C, but using primers specific for the *MFGE8* gene. (**C**) Exogenous expression of FLAG-tagged PC4 wild-type (wt) or SMYD interaction-deficient Q65/R75 mutant (mt) in PC4-depleted HCT116 colon cancer cells was confirmed by immunoblotting. (**D**) ChIP-qPCR assays were done with PC4-depleted HCT116 cells complemented with PC4 wild-type (wt) or mutant (mt) as in (**B**). The vertical red bar in (**B**) and (**D**) represents the putative SMYD3 binding site.

Figure S9. RT-PCR and ChIP of the PC4 target gene *SMUG1*.
(**A**) SMYD3-depleted HCT116 cells were transfected with SMYD3 wild-type (wt) or K78/D82/R85 mutant (mt), and the expression of the known PC4 target gene *SMUG1* was determined by qRT-PCR. (**B**) SMYD3-depleted HCT116 cells were complemented with SMYD3 wild-type (wt) or K78/D82/R85 mutant (mt) as in (**A**), and the levels of SMYD3, PC4 and H3K4me3 along the *SMUG1* gene were determined by ChIP-qPCR. (**C**) Wild type (wt) or Q65/R75-mutated (mt) PC4 was expressed in PC4-depleted HCT116 cells, and *SMUG1* gene expression was determined by qRT-PCR. (**D**) ChIP signals for SMYD3, PC4 and H3K4me3 along the *SMUG1* gene were determined as indicated.

Figure S10. dCas9-based activation of SMYD3 target genes.
qRT-PCR experiments were performed as in Figure 5C, but using sgRNAs specific for the *MFGE8* gene.

Figure S11. dCas9-based accumulation of SMYD3 and H3K4me3 at target genes.
ChIP-qPCR experiments were performed as in Figure 6, but using sgRNAs and primers specific for the *MFGE8* gene.

Supplementary Table S2. sgRNA target sites and oligo primer sequences.

Name	Target site	Forward (5'-3')	Reverse (5'-3')
FNBP1-G1	CCCGGGGGAGGGGGCCGGGC	ACACCCCCGGGGGAGGGGGCCGGGCG	AAAACGCCCGGCCCCCTCCCCCGGGG
FNBP1-G2	CCGCCCCTCCCCCGCTCTCC	ACACCCCGCCCCTCCCCCGCTCTCCG	AAAACGGAGAGCGGGGGAGGGGCGGG
FNBP1-G3	CCGAGCTCTGGGTGAGTGAG	ACACCCCGAGCTCTGGGTGAGTGAGG	AAAACCTCACTCACCCAGAGCTCGGG
FNBP1-G4	CTCGCGGCCTGGGGAGGAGG	ACACCCTCGCGGCCTGGGGAGGAGGG	AAAACCCTCCTCCCCAGGCCGCGAGG
MFGE8-G1	AACCAATTCCCTCCCAGCCG	ACACCAACCAATTCCCTCCCAGCCGG	AAAACCGGCTGGGAGGGAATTGGTTG
MFGE8-G2	GCCAGCTTGGGCGGAGCGCA	ACACCGCCAGCTTGGGCGGAGCGCAG	AAAACTGCGCTCCGCCCAAGCTGGCG
MFGE8-G3	TAAGGGAAGGGCCGGGGAGT	ACACCTAAGGGAAGGGCCGGGGAGTG	AAAACACTCCCCGGCCCTTCCCTTAG
MFGE8-G4	GGAAGAGGGCTGCCTGCTGA	ACACCGGAAGAGGGCTGCCTGCTGAG	AAAACTCAGCAGGCAGCCCTCTTCCG

Supplementary Table S3. Primer sequences for qRT-PCR.

Primers	Forward (5'-3')	Reverse (5'-3')
ACTB	TCACCGAGCGCGGCT	TAATGTCACGCACGATTTCCC
ARAF	GAATGAGATGCAGGTGCTCA	CCACATGCAGGTGATGGTAG
GRINA	CGGGAGAATGTCTGGACCTA	CTGCATGGAGAAGATGACGA
MAP2K3	AGCTACCTGGAGCTGATGGA	AGCTTATGGTGTGGGTGAGC
MAPK3	ACAGTCTCTGCCCTCCAAGA	CTCATCCGTCGGGTCATAGT
P4HB	CATCGTGAACTGGCTGAAGA	CTCCACGTCCTTGAAGAAGC
PLCG2	AACCAACCAGCAAAACCAAG	TTTGTCCCTTTGGGTAGACG
DERL1	GCTTTCGACTTGGAGACCAG	TGTTAGCCAGAACGCAGTTG
FNBP1	TGGAGGAAAGGAGGATTGTG	CGCTTCATTGGCTGAGTGTA
FTL	AGGCCCTTTTGGATCTTCAT	CAGGTGGTCACCCATCTTCT
GAL	AAGGAAAAACGAGGCTGGAC	GGACCTGTCAAAGCTTCCTG
MFGE8	ACCTGTTTGAGACCCCTGTG	GGTTCCAGCTGAAGAGATGC
PDLIM7	CAGCTTCTGCCTACCTCACC	AAAGAGGGAAGGCAGTGTCA
SLC9A1	TCTTCACCGTCTTTGTGCAG	AAGGTGGTCCAGGAACTGTG
SNCA	TGTGCCCAGTCATGACATTT	CCACAAAATCCACAGCACAC
TOB1	ACAGCCCCCTTAACCTCAGT	GCCCGTGCATTTTAACTTGT
VGF	AGCCTCCTCCTCTCAGCTCT	GAAAAGCTCTCCCTCGTCCT
WNT3A	CAAGATTGGCATCCAGGAGT	ATGAGCGTGTCACTGCAAAG
KRT81	TAGGCACCCCAACTCAAGTC	AAGTGGGGGATCACACAGAG
GAPDH	GGCCTCCAAGGAGTAAGACC	AGGGGAGATTCAGTGTGGTG
SMUG1	AGCTGGGGGAAGGATGTAGT	GCCAACATGGTGAAACACTG

Supplementary Table S4. Primer sequences for qPCR.

Primers	Forward (5'-3')	Reverse (5'-3')
PDLIM7-TSS	GAAAGTCTAGTGGGCGTGGT	AGTGCCCTTTGTGGTCCTC
WNT3A-TSS	CACAGACCAGGAGCGAGAG	GTGTCGGTGTGTCTGAGGAC
KRT81-TSS	GCCAGGCTCTACTCCTCCTT	GGGGAGTCAGCATGTTTCAT
FNBP1-A	GCTGTCCCTTCCTCTTCCTC	TTTGGAAACGGTACATGCAA
FNBP1-B	GGGGAAAGGGCTAGTGATTC	CAGCGCTCATTTTATCAGCA
FNBP1-C	TACTGCGAGCAGACAGTGGT	CAAAATGGCCCGAGGAAG
FNBP1-D	CGAGCTCTGGGTGAGTGAG	TCGCCTCCGAAGGACAAG
FNBP1-E	CAAAGGGTGTGGAGGAGAAA	TATTGCCCTCAGGATTTTGG
FNBP1-F	GTGGCTCAGGCCTATATTGC	TCCAGATATTCCCCAAGCTG
MFGE8-A	CCACCATGCCTGGTTACTTT	GAAAGGGAGCTTCCTCAACC
MFGE8-B	GCACCCAGCCAAAACACTAT	CTGTTTGCCGAAAGGGAATA
MFGE8-C	CCCAACAGACTCAAGACTCG	CCTCTGGGACCGGAATAAAT
MFGE8-D	GGCTGGACAGTTCATTGGAT	GTCAGGAGACTCCCATCAGC
MFGE8-E	CCAAGCCTCTGTCTGTGTCA	GGGCCTCAGGAGCTTAAAGT
MFGE8-F	CCCACTCCCATCTCACACTT	TGGGAGGGATCTGAAGAATG
SMUG1-A	GGCTTCATGAGGTGGATGAT	GGTGCAGTAGGAGCTTCAGG
SMUG1-B	GGCTCTGGGACTTAGCAGTG	TCTCCCGCTCTGCTGTCTAT
SMUG1-C	ACAGGCTGTGGCTTTTCACT	GCTAAGGACAGTGGGAGTCG
SMUG1-D	CACCATGCCCAGCTAATTTT	GACTTCGAGACCAGCCTGAC
SMUG1-E	ATGTCAAAGGTGGGCTCAAG	GGGGAAGTGGGAGAGAAAAG
SMUG1-F	ACGGGACCTACTTGTGTTGG	CCCAACCTGTCCACATATCC

Supplementary Table S5. GSEA of 9 gene sets significantly enriched in the SMYD3-knock down condition (normalized (NOM) p<0.01 and false discovery rate (FDR) q<0.05).

NAME	SIZE	ES	NES	NOM p-val	FDR q-val
BENPORATH PROLIFERATION	78	0.423409	4.035999	0	0
POOLA INVASIVE BREAST CANCER UP	83	0.252012	2.556257	0	3.18E-04
ROSTY CERVICAL CANCER PROLIFERATION CLUSTER	84	0.544624	5.226195	0	0
CHIANG LIVER CANCER SUBCLASS PROLIFERATION UP	85	0.397859	4.045592	0	0
PUIFFE INVASION INHIBITED BY ASCITES DN	65	0.326433	2.170377	0.005865	0.015505
PEART HDAC PROLIFERATION CLUSTER DN	40	0.352734	1.989805	0.013921	0.020701
PEART HDAC PROLIFERATION CLUSTER UP	56	0.305071	1.880649	0.012019	0.029155
PUIFFE INVASION INHIBITED BY ASCITES UP	35	0.296035	1.724907	0.010392	0.03608
WANG TUMOR INVASIVENESS UP	147	0.195778	1.674531	0.014035	0.04197

Supplementary Table S6. Molecular and cellular function analysis of IPA in SMYD3 knockdown repressed genes.

Name	Number of genes	p-value
Cell death and Survival	77	5.98E-06 - 1.27E-02
Cellular Growth and Proliferation	72	4.45E-04 - 1.23E-02
Cellular Function and Maintenance	71	1.03E-04 - 1.37E-02
Cellular Assembly and Organization	54	1.50E-04 - 1.41E-02
Cellular Development	51	4.45E-04 - 1.23E-02
Molecular Transport	50	2.79E-04 - 1.41E-02
Small Molecule Biochemistry	41	3.54E-05 - 1.41E-02
Cell-To-Cell Signaling and Interaction	31	4.45E-04 - 1.41E-02
Cellular Compromise	26	1.03E-04 - 1.41E-02
Carbohydrate Metabolism	23	9.58E-05 - 1.23E-02
Post-Translational Modification	14	3.54E-05 - 1.23E-02
Protein Trafficking	10	2.79E-04 - 1.23E-02
Amino Acid Metabolism	5	3.54E-05 - 1.23E-02

Supplementary Table S7. Molecular and cellular function analysis of IPA in SMYD3 knockdown activated genes.

Name	Number of genes	p-value
Cell death and Survival	31	2.12E-06 – 2.18E-02
Cell Cycle	21	3.00E-05 – 2.24E-02
Cellular Growth and Proliferation	20	3.44E-05 – 2.24E-02
DNA Replication, Recombination, and Repair	15	4.49E-05 – 2.18E-02
Cellular Development	31	1.13E-04 – 2.30E-02
RNA Post-Transcriptional Modification	7	1.99E-04 – 1.69E-02
Cellular Compromise	5	1.01E-03 – 1.35E-02
Cell Morphology	20	1.87E-03 – 1.68E-02
Cellular Function and Maintenance	12	2.99E-03 – 2.02E-02
Cellular Movement	13	3.10E-03 – 2.27E-02
Immune Cell Trafficking	8	3.10E-03 – 1.58E-02
Inflammatory Response	8	3.10E-03 – 1.58E-02
Amino Acid Metabolism	1	3.40E-03 – 3.40E-03
Carbohydrate Metabolism	4	3.40E-03 – 1.69E-02

Figure S1

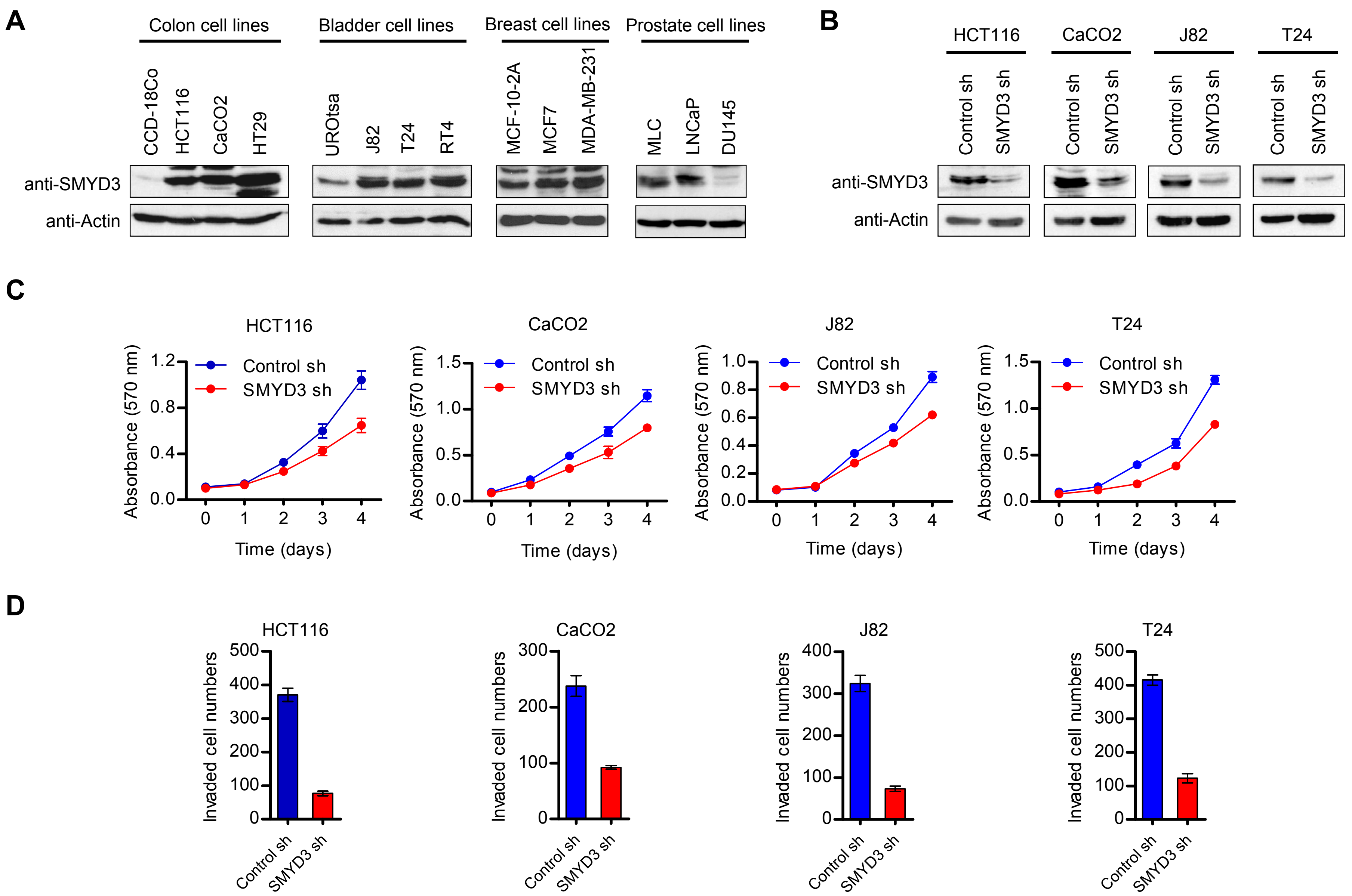
A
Colon cell lines
Bladder cell lines
Breast cell lines
Prostate cell lines
CCD-18Co
HCT116
CaCO2
HT29
UROtsa
J82
T24
RT4
MCF-10-2A
MCF7
MDA-MB-231
MLC
LNCaP
DU145
anti-SMYD3
anti-Actin
B
HCT116
CaCO2
J82
T24
Control sh
SMYD3 sh
anti-SMYD3
anti-Actin
C
HCT116
CaCO2
J82
T24
Absorbance (570 nm)
Control sh
SMYD3 sh
Time (days)
D
HCT116
CaCO2
J82
T24
Invaded cell numbers
Control sh
SMYD3 sh

Figure S2

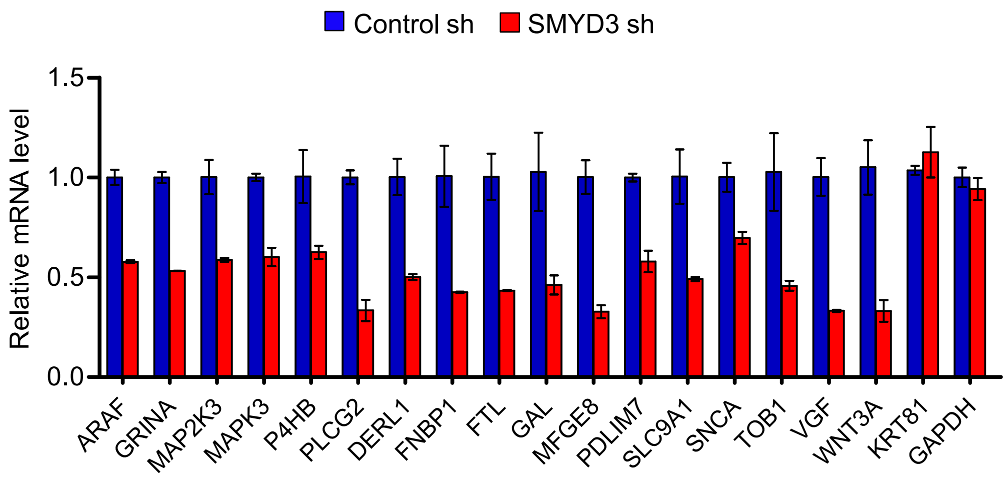
Control sh
SMYD3 sh
1.5
1.0
0.5
0.0
Relative mRNA level
ARAF
GRINA
MAP2K3
MAPK3
P4HB
PLCG2
DERL1
FNBP1
FTL
GAL
MFGE8
PDLIM7
SLC9A1
SNCA
TOB1
VGF
WNT3A
KRT81
GAPDH

Figure S3

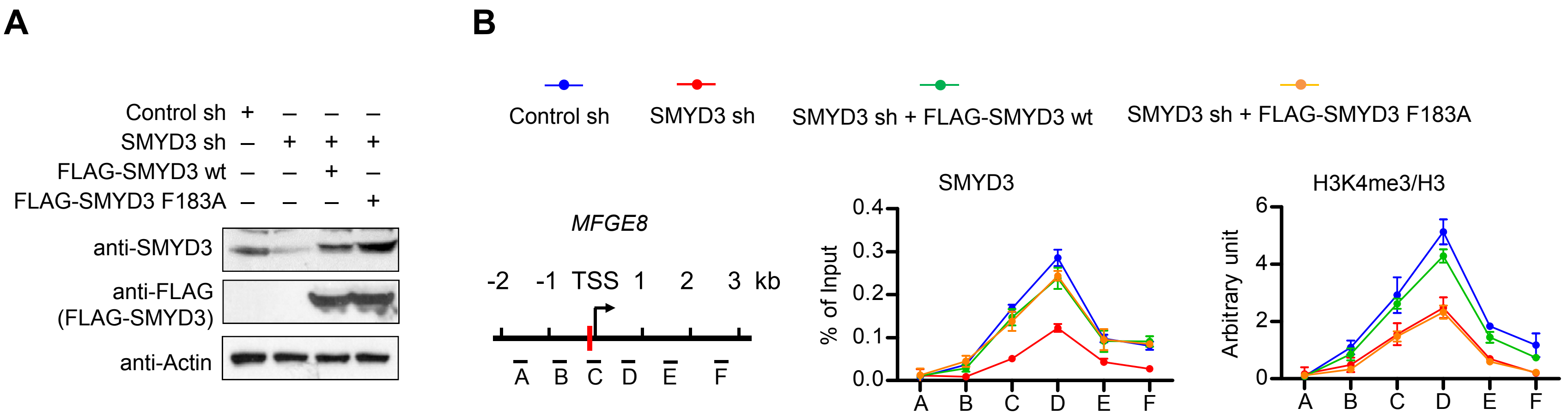
A
Control sh + – – –
SMYD3 sh – + + +
FLAG-SMYD3 wt – – + –
FLAG-SMYD3 F183A – – – +
anti-SMYD3
anti-FLAG
(FLAG-SMYD3)
anti-Actin
B
Control sh
SMYD3 sh
SMYD3 sh + FLAG-SMYD3 wt
SMYD3 sh + FLAG-SMYD3 F183A
MFGE8
-2 -1 TSS 1 2 3 kb
A B C D E F
SMYD3
% of Input
0.0 0.1 0.2 0.3 0.4
H3K4me3/H3
Arbitrary unit
0 2 4 6

Figure S4

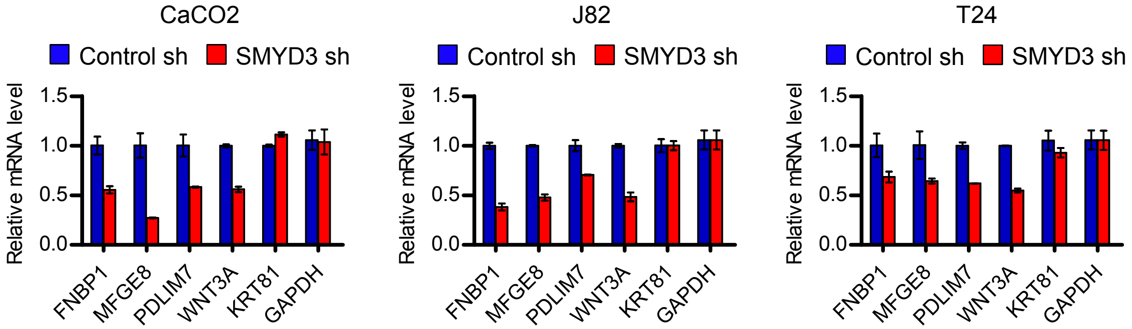
CaCO2
Control sh
SMYD3 sh
Relative mRNA level
1.5
1.0
0.5
0.0
FNBP1
MFGE8
PDLIM7
WNT3A
KRT81
GAPDH
J82
Control sh
SMYD3 sh
Relative mRNA level
1.5
1.0
0.5
0.0
FNBP1
MFGE8
PDLIM7
WNT3A
KRT81
GAPDH
T24
Control sh
SMYD3 sh
Relative mRNA level
1.5
1.0
0.5
0.0
FNBP1
MFGE8
PDLIM7
WNT3A
KRT81
GAPDH

Figure S5

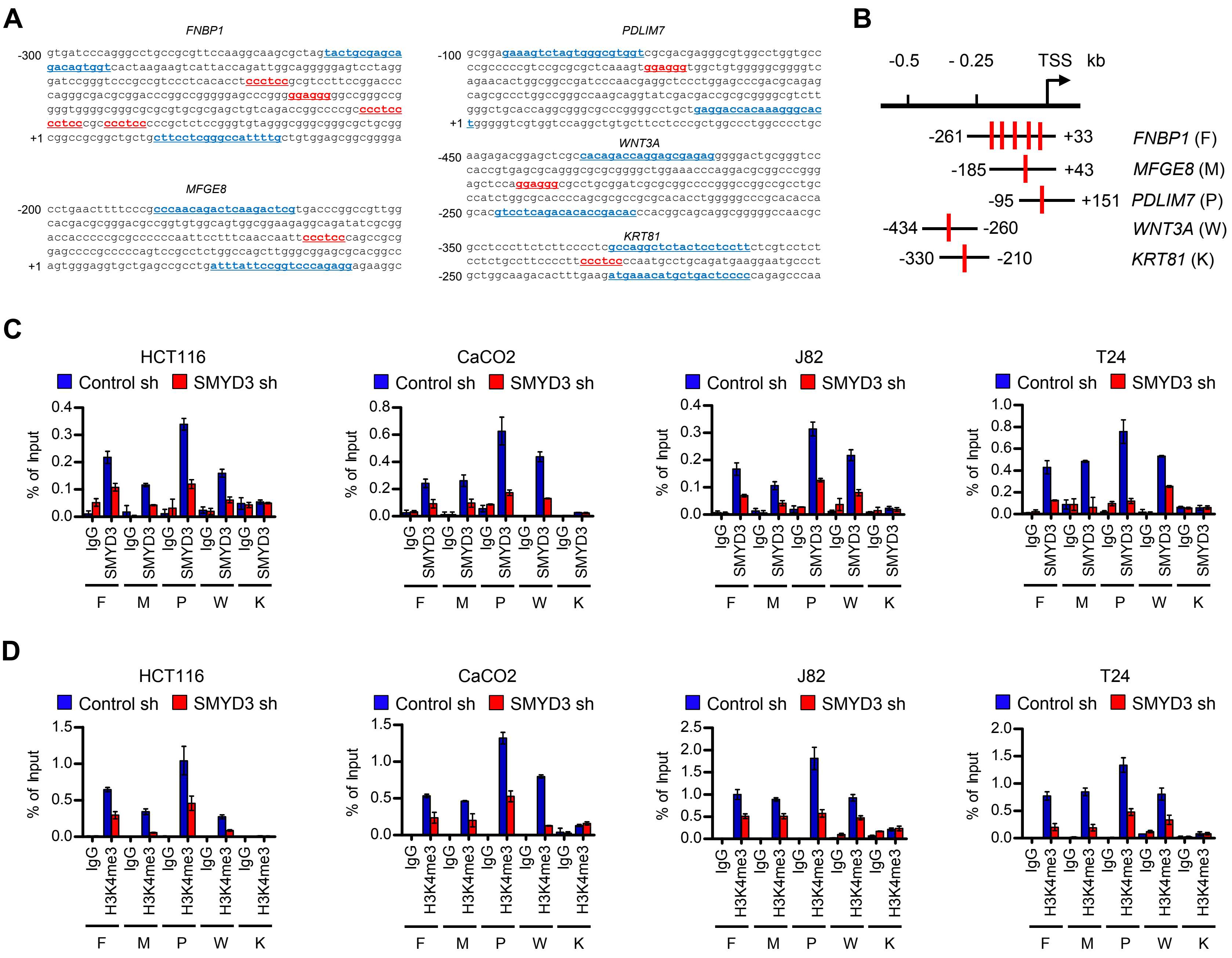
A
FNBP1
-300 gtgatcccagggcctgccgcgttccaaggcaagcgctagtactgcgagca
gacagtggtcactaagaagtcattaccagattggcaggggagtcctagg
gatccgggtcccgccgtccctcacacctccctccgcgtccttccggaccc
cagggcgacgcggacccggccgggggagcccgggggaggggccgggccg
gggtggggcgggcgcgcgtgcgcgagctgtcagaccggccccgcccctcc
cctcccgcccctcccccgctctccgggtgtagggcgggcgggcgctgcgg
+1 cggccgcggctgctgcttcctcgggccattttgctgtggagcggcgggga
MFGE8
-200 cctgaactttttcccgcccaacagactcaagactcgtgacccggccgttgg
cacgacgcgggacgccggtgtggcagtggcggaagaggcagatatcgcgg
accacccccgcgccccccaattcctttcaaccaattccctcccagccgcg
gagccccgcccccagtccgcctctggccagcttgggcggagcgcacggcc
+1 agtgggaggtgctgagccgcctgatttattccggtcccagaggagaaggc
PDLIM7
-100 gcggagaaagtctagtgggcgtggtcgcgacgagggcgtggcctggtgcc
ccgcccccgtccgcgcgctcaaagtggagggtggctgtgggggcggggtc
agaacactggcggccgatcccaacgaggctccctggagcccgacgcagag
cagcgccctggccgggccaagcaggtatcgacgaccgcgcggggcgtctt
gggctgcaccaggcgggcgcccggggcctgctgaggaccacaaagggcac
+1 tgggggtcgtggtccaggctgtgcttcctcccgctggccctggcccctgc
WNT3A
-450 aagagacggagctcgccacagaccaggagcgagagggggactgcgggtcc
caccgtgagcgcagggcgcgcggggctggaaacccaggacgcggcccggg
agctccaggagggcgcctgcggatcgcgcggccccgggccggccgcctgc
ccatctggcgcaccccagcgcgccgcgcacacctgggggcccgcacacca
-250 gcacgtcctcagacacaccgacacccacggcagcaggcgggggccaacgc
KRT81
-350 gcctcccttctcttcccctcgccaggctctactcctccttctcgtcctct
cctctgccttccccttccctccccaatgcctgcagatgaaggaatgccct
-250 gctggcaagacactttgaagatgaaacatgctgactccccagagcccaa
B
-0.5
- 0.25
TSS
kb
-261
+33
FNBP1 (F)
-185
+43
MFGE8 (M)
-95
+151
PDLIM7 (P)
-434
-260
WNT3A (W)
-330
-210
KRT81 (K)
C
HCT116
CaCO2
J82
T24
Control sh
SMYD3 sh
% of Input
IgG
SMYD3
F
M
P
W
K
D
HCT116
CaCO2
J82
T24
Control sh
SMYD3 sh
% of Input
IgG
H3K4me3
F
M
P
W
K

Figure S6

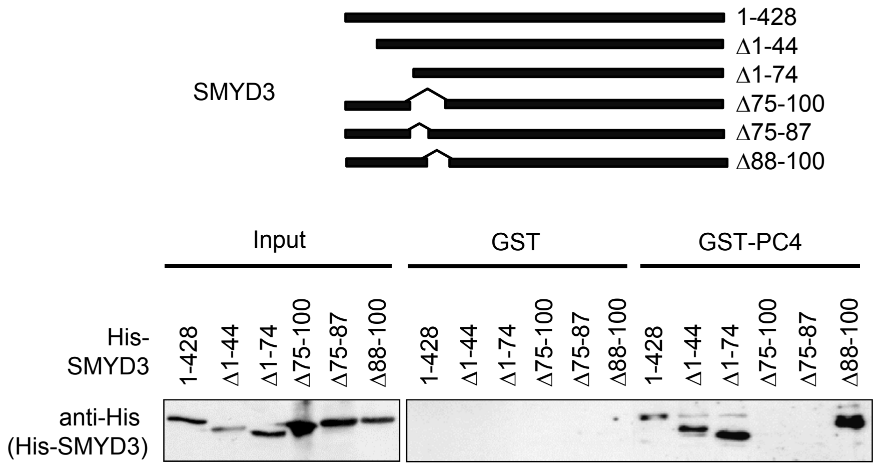

SMYD3
1-428
Δ1-44
Δ1-74
Δ75-100
Δ75-87
Δ88-100
Input
GST
GST-PC4
His-
SMYD3
1-428
Δ1-44
Δ1-74
Δ75-100
Δ75-87
Δ88-100
anti-His
(His-SMYD3)

Figure S7

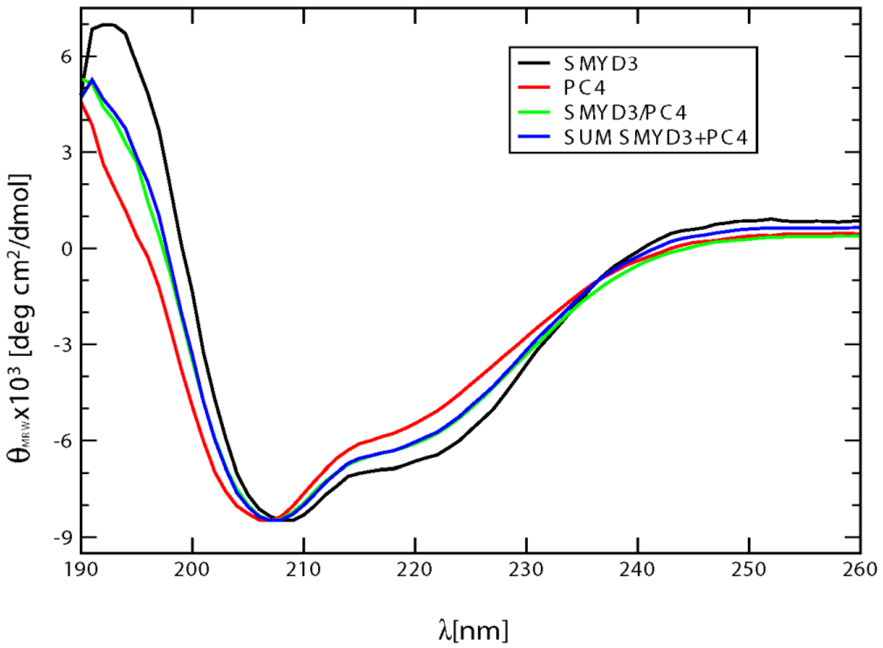
SMYD3
PC4
SMYD3/PC4
SUM SMYD3+PC4
6
3
0
-3
-6
-9
190
200
210
220
230
240
250
260
θMRW x10³ [deg cm²/dmol]
λ[nm]

Figure S8

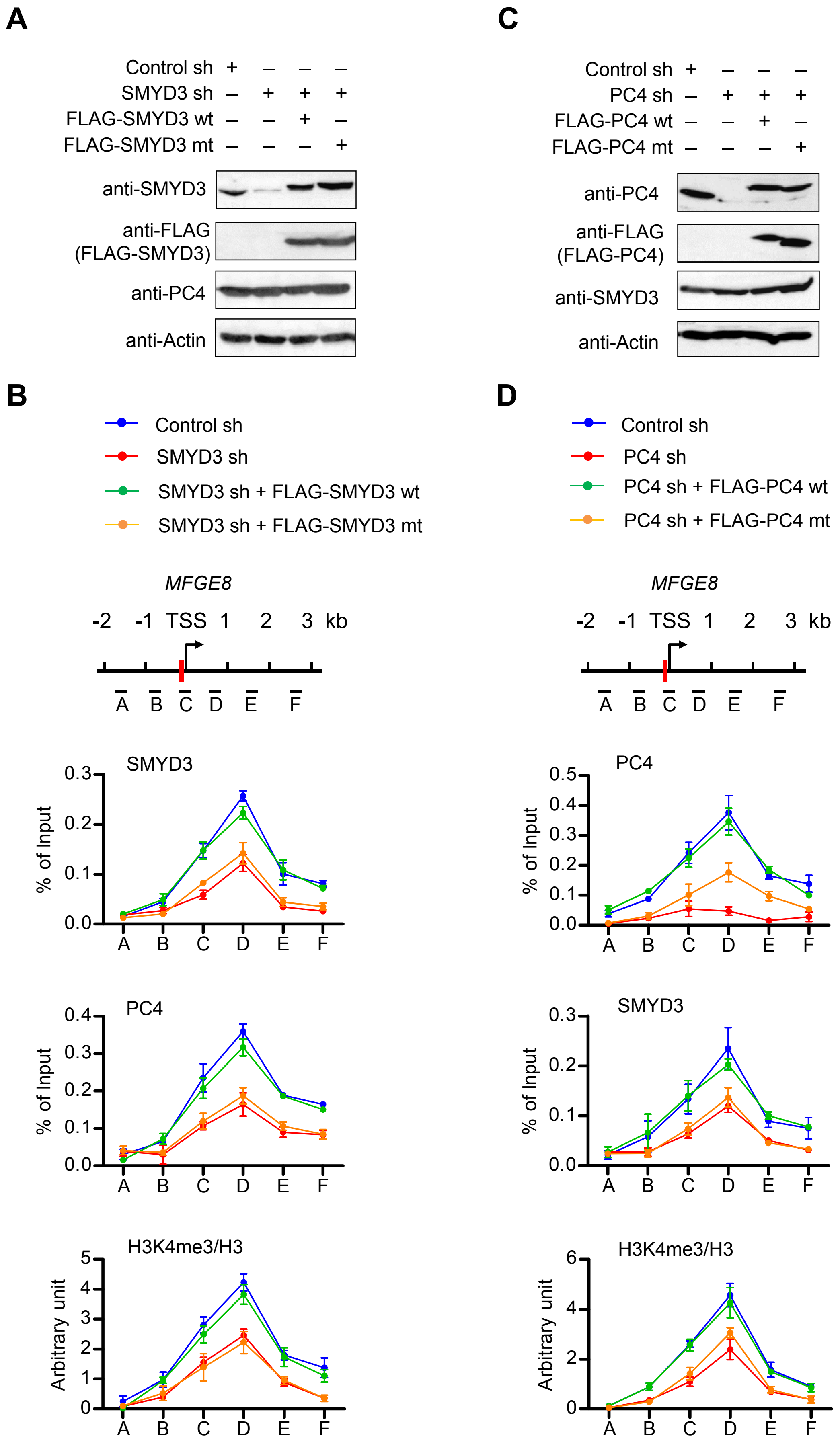
A
Control sh + – – –
SMYD3 sh – + + +
FLAG-SMYD3 wt – – + –
FLAG-SMYD3 mt – – – +
anti-SMYD3
anti-FLAG (FLAG-SMYD3)
anti-PC4
anti-Actin
C
Control sh + – – –
PC4 sh – + + +
FLAG-PC4 wt – – + –
FLAG-PC4 mt – – – +
anti-PC4
anti-FLAG (FLAG-PC4)
anti-SMYD3
anti-Actin
B
Control sh
SMYD3 sh
SMYD3 sh + FLAG-SMYD3 wt
SMYD3 sh + FLAG-SMYD3 mt
MFGE8
-2 -1 TSS 1 2 3 kb
A B C D E F
SMYD3
PC4
H3K4me3/H3
% of Input
Arbitrary unit
D
Control sh
PC4 sh
PC4 sh + FLAG-PC4 wt
PC4 sh + FLAG-PC4 mt
MFGE8
-2 -1 TSS 1 2 3 kb
A B C D E F
PC4
SMYD3
H3K4me3/H3
% of Input
Arbitrary unit

Figure S9

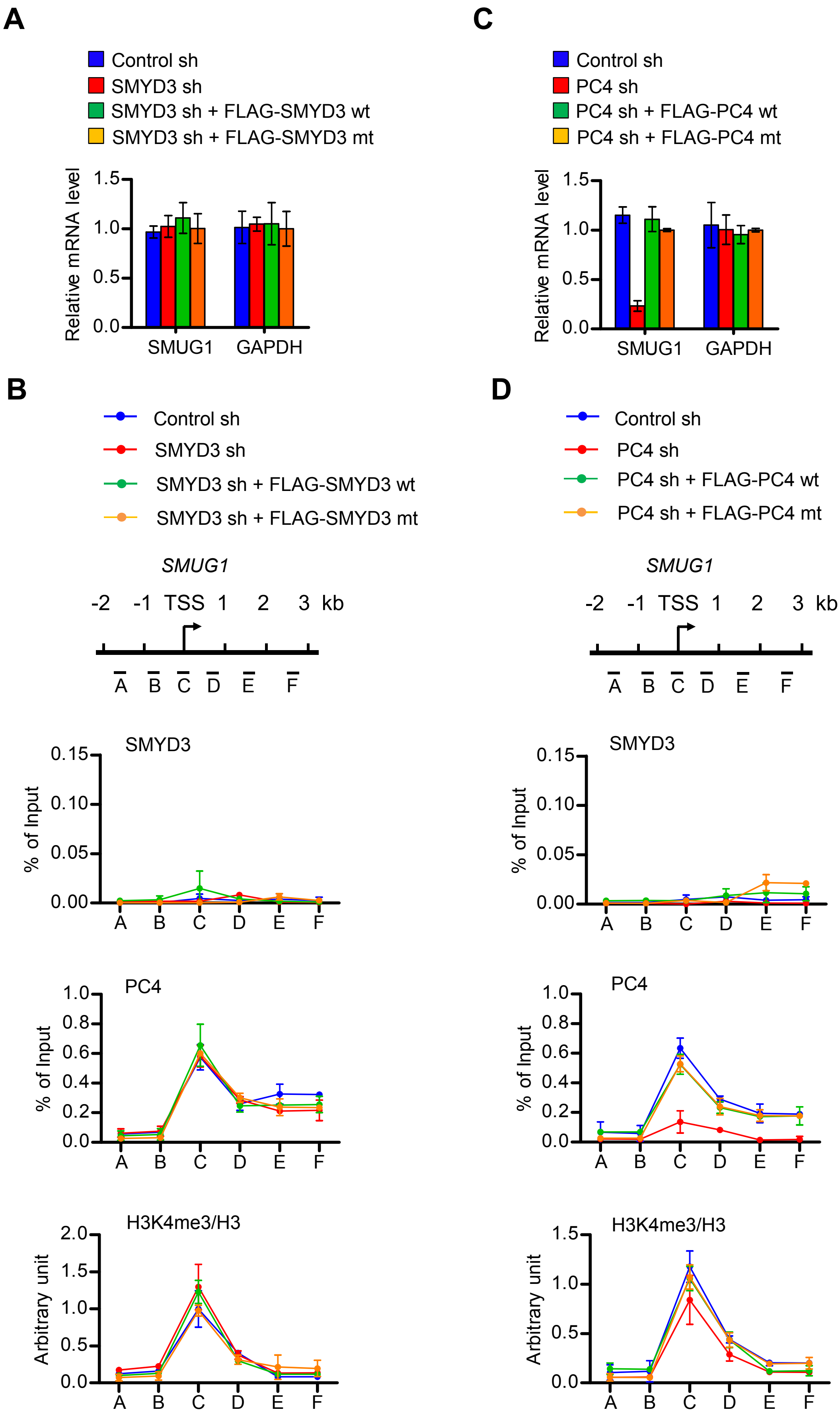

A
Control sh
SMYD3 sh
SMYD3 sh + FLAG-SMYD3 wt
SMYD3 sh + FLAG-SMYD3 mt
Relative mRNA level
1.5
1.0
0.5
0.0
SMUG1
GAPDH
C
Control sh
PC4 sh
PC4 sh + FLAG-PC4 wt
PC4 sh + FLAG-PC4 mt
Relative mRNA level
SMUG1
GAPDH
B
Control sh
SMYD3 sh
SMYD3 sh + FLAG-SMYD3 wt
SMYD3 sh + FLAG-SMYD3 mt
SMUG1
-2 -1 TSS 1 2 3 kb
A B C D E F
SMYD3
% of Input
0.15
0.10
0.05
0.00
PC4
1.0
0.8
0.6
0.4
0.2
0.0
H3K4me3/H3
Arbitrary unit
2.0
1.5
1.0
0.5
0.0
D
Control sh
PC4 sh
PC4 sh + FLAG-PC4 wt
PC4 sh + FLAG-PC4 mt
SMUG1
-2 -1 TSS 1 2 3 kb
A B C D E F
SMYD3
PC4
H3K4me3/H3
Arbitrary unit

Figure S10

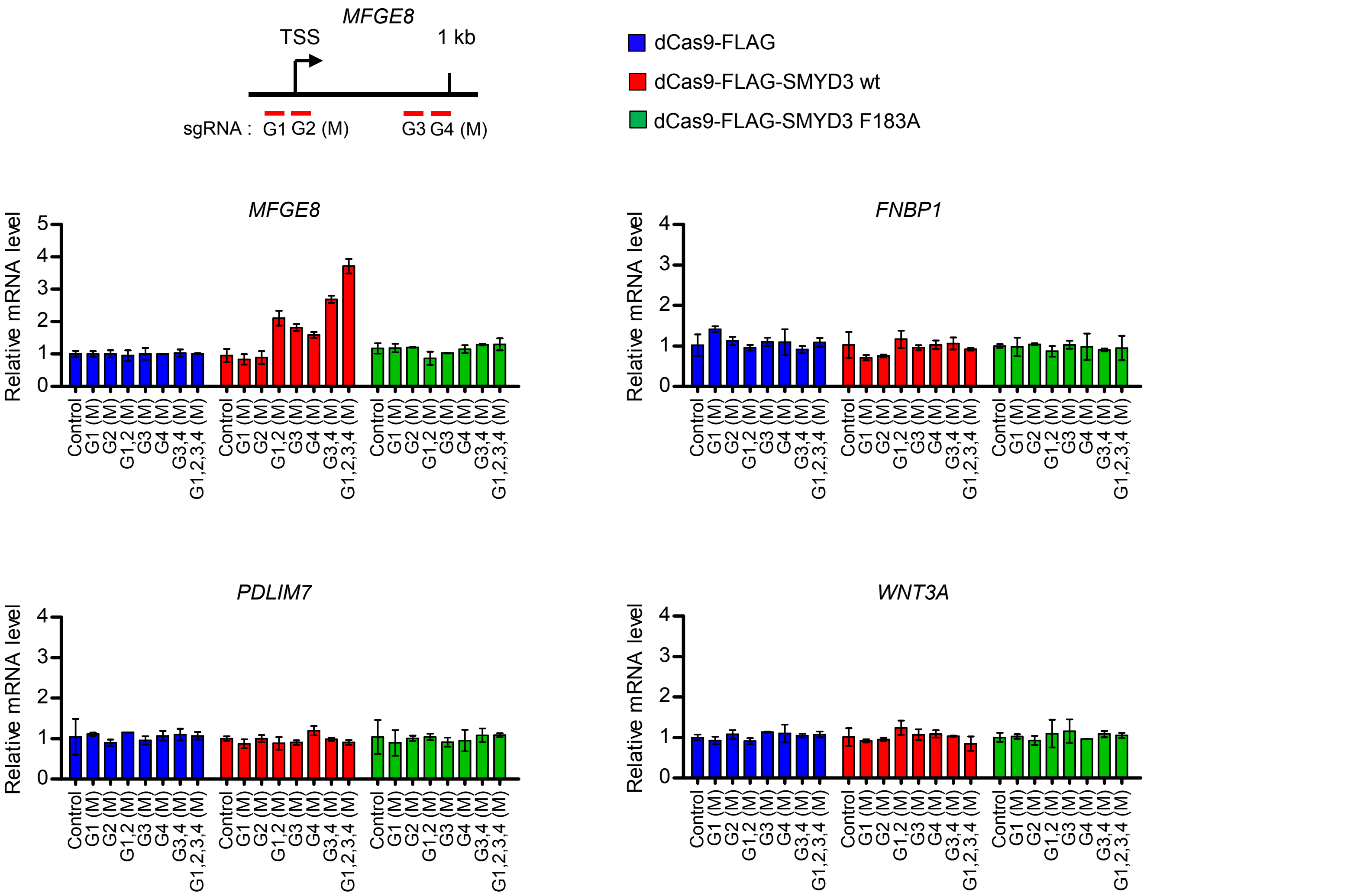
MFGE8
TSS
1 kb
sgRNA : G1 G2 (M) G3 G4 (M)
dCas9-FLAG
dCas9-FLAG-SMYD3 wt
dCas9-FLAG-SMYD3 F183A
MFGE8
FNBP1
PDLIM7
WNT3A
Relative mRNA level
Control
G1 (M)
G2 (M)
G1,2 (M)
G3 (M)
G4 (M)
G3,4 (M)
G1,2,3,4 (M)

Figure S11

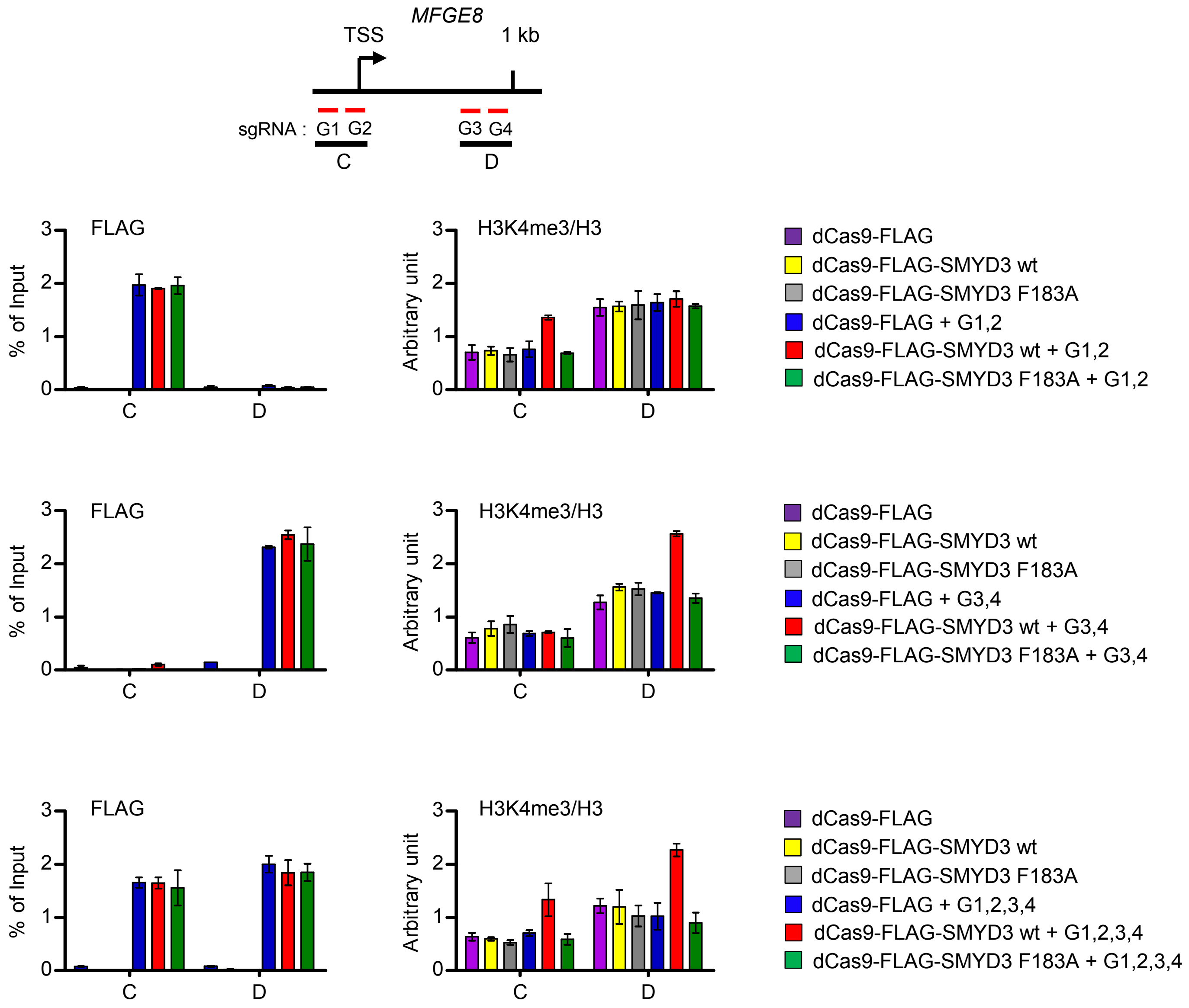
MFGE8
TSS
1 kb
sgRNA : G1 G2 G3 G4
C
D
FLAG
% of Input
H3K4me3/H3
Arbitrary unit
dCas9-FLAG
dCas9-FLAG-SMYD3 wt
dCas9-FLAG-SMYD3 F183A
dCas9-FLAG + G1,2
dCas9-FLAG-SMYD3 wt + G1,2
dCas9-FLAG-SMYD3 F183A + G1,2
dCas9-FLAG + G3,4
dCas9-FLAG-SMYD3 wt + G3,4
dCas9-FLAG-SMYD3 F183A + G3,4
dCas9-FLAG + G1,2,3,4
dCas9-FLAG-SMYD3 wt + G1,2,3,4
dCas9-FLAG-SMYD3 F183A + G1,2,3,4
